# Metagenomic profiling of gut microbiome signatures across liver disease stages and HCV-related hepatocellular carcinoma in Egyptian patients

**DOI:** 10.3389/fmicb.2026.1758563

**Published:** 2026-04-28

**Authors:** Marwa Zahra, Amged Ouf, Hassan M. E. Azzazy, Ahmed Moustafa

**Affiliations:** 1 Graduate Biotechnology Program, Biology Department, School of Sciences & Engineering, The American University in Cairo, New Cairo, Egypt; 2 Graduate Biotechnology Program, Chemistry Department, School of Sciences & Engineering, The American University in Cairo, New Cairo, Egypt; 3 Generations Genetics, New Cairo, Egypt

**Keywords:** Egypt, gut, HCV, hepatocellular carcinoma, metagenomics, microbiome, whole-metagenome sequencing

## Abstract

**Introduction:**

Dysbiosis in the gut microbiome, particularly concerning the synchronous crosstalk between the gut and the liver, has been associated with various diseases. This study examines the gut microbiome's role in liver diseases among Egyptian patients, with a focus on the hepatitis C virus (HCV) and hepatocellular carcinoma (HCC), both of which are highly prevalent in Egypt.

**Methods:**

Utilizing shotgun metagenomic sequencing, we analyzed microbial gene catalogs and taxonomic profiles from 46 Egyptian patients categorized into five groups: healthy individuals, liver disease patients of different etiologies, post-HCV, treated HCV, and HCV-HCC patients.

**Results:**

Healthy and treated HCV patients exhibited distinct microbial profiles characterized by an abundance of beneficial bacteria, *Faecalibacterium* and *Bifidobacterium* (*p* < 0.05), associated with anti-inflammatory short-chain fatty acid production. Conversely, liver disease and HCC patients displayed increased pathogenic bacteria, *Escherichia* (*p* < 0.05), and genes linked to inflammation and oncogenesis, including lipopolysaccharide biosynthesis.

**Discussion:**

These findings suggest a dominance of *Faecalibacterium* in healthy Egyptians, likely attributable to traditional dietary patterns, and cytochrome P450 genes as potential HCC biomarkers, possibly connected to aflatoxin exposure. Treated HCV patients showed significant microbiome recovery, reflecting effective antiviral therapy. These findings emphasize that Egypt-specific factors, such as persistent resistance genes post-HCV due to antibiotic use and the prominence of bile acid metabolism genes, are influenced by high HCV prevalence and environmental exposures like aflatoxins. Taken together, the results highlight the need for region-specific microbiome research priorities in Egypt and underscore how local dietary, clinical, and environmental factors may shape future objectives in understanding liver disease pathogenesis and prevention.

## Background

An understanding of the microbial environment associated with the etiologies of HCC provides new insight into the root causes of HCC. The microbiota of an individual is similar to a microbial “fingerprint” that gives a message about the health status of the organism it survives with. The gut is composed of various bacteria in addition to archaea, viruses, and protists that play a role in maintaining the homeostasis and vital functions of the healthy host by generating active metabolites (El-Mowafy et al., 2021). Four major bacterial phyla, Bacteroidetes, Firmicutes, Actinobacteria, and Proteobacteria, play a predominant role in gut health (El-Mowafy et al., 2021).

The liver, due to its anatomical location, is very closely related to the gut. The majority of the liver's blood and nutritional supply is derived from the gut through the portal vein. The link between the gut microbiota, systemic circulation, the biliary tract, and the liver represents the gut-liver axis. The communication and transport of gut microbiota and their toxic byproducts to the liver, such as peptidoglycans, endotoxins, or intact bacteria, may disrupt the metabolic functions of the liver. While the reverse is also true, as bile acids are generated in the liver, the conversion and alteration of bile acids and their release into the intestine disrupt the regulation and communication of the gut-liver axis. Gut microbiota dysbiosis, in turn, is essential to understanding liver diseases, especially liver cancer.

Hepatocellular Carcinoma (HCC) is unlike any other cancer in that it stems from a multifaceted organ, the liver. This end-stage disease arises from many etiologies of the liver, all ultimately with the same outcome. Globally, HCC registers as the sixth most diagnosed cancer and the fourth deadliest, with over 900,000 new cases and 830,000 deaths annually (Loomba et al., 2017). In 2018, Egypt ranked as the second-highest country for liver cancer risk globally, trailing only Mongolia. Over 90% of HCC cases arise in the context of chronic liver disease, with cirrhosis from any cause emerging as the most significant predisposing factor (Rashed et al., 2020). Many risk factors influence the development of HCC, including viral hepatitis infections, cirrhosis of the liver, cryptogenic and autoimmune hepatitis, as well as environmental and chemical toxin exposures, lifestyle-related factors, and genetic predispositions.

Although the mechanisms by which microbial dysbiosis participates in disease progression are not fully understood, they can be classified into three categories. The alteration of beneficial microbial environments marks disease progression via dysbiosis, the prevalence of harmful or pathogenic microorganisms, and the modification of total microbial diversity. In viral hepatitis, the immune system is evaded via many mechanisms, including dysbiosis of the intestinal microbiota. These translocations of the intestinal microbiota impair the primary barrier, allowing for the rapid growth of pathogenic bacteria and the abnormal regulation of immune cells, which result in severe intestinal inflammation.

One of the major etiological agents of HCC is viral hepatitis infection, which results in severe liver complications, marked by liver cirrhosis, liver cancer, liver failure, and death. There have been specific bacterial taxa identified that have an impact on viral replication as well as host cell and virus interaction, such as the family *Enterobacteriaceae*. During viral hepatitis, *Enterobacteriaceae* prevail, especially the species *Enterococcus faecalis, Escherichia coli*, and *Faecalibacterium prausnitzii*. In contrast, species such as lactic acid-producing bacteria, such as *Lactobacillus* and *Pediococcus*, are reduced. The fluctuations of these bacterial communities lead to the deprivation of short-chain fatty acids, loss of pH control of the colon, hyperammonemia, inflammation, endotoxemia, and the growth of pathogenic bacteria. These, in turn, worsen the condition of the disease and the progression to end-stage HCC.

Identifying microbial signatures for HCC risk and progression could enhance early detection and prognosis, but current data lack specificity across etiologies. While associations between gut microbiota and HCC are well-documented, causal relationships remain elusive, necessitating the need for etiologically specific mechanisms that explore disease progression.

Using shotgun metagenomic sequencing to identify gut microbiota for patients in five categories—normal, HCV, liver disease patients of etiologies such as cryptogenic and autoimmune hepatitis, HCV-treated patients, and HCC patients in Egypt could provide more insight into how the microbial communities fluctuate during disease progression. Prominent microbial taxa could be used as biomarkers for specific disease stages, and treatment strategies can be developed via a better understanding of beneficial taxa that emerge after disease treatment.

## Methods

### Study design and subjects

Stool samples were collected from patients in the internal medicine ward at the Theodore Bilharz Institute Hospital, Cairo, Egypt. Ethical approval for collecting and analyzing patient stool samples was obtained under reference number 2020–2021-056 from the Institutional Review Board at The American University in Cairo. All participants provided written and signed consent before sample collection. A total of 46 samples were collected from patients of four specific experimental groups; these included 12 samples for patients with active HCV viral load, 10 samples for HCV-treated patients PCR PCR-negative, six samples for HCV/HCC patients, eight samples for patients with liver disease of different etiologies, and 10 samples for the healthy control group. Exclusion criteria included inflammatory bowel syndrome, colorectal cancers, or any use of systemic antibiotics, probiotics, or prebiotics during or 3 months before the study initiation. Essential liver functions were collected from the patient medical records. Other necessary information about other heart, chest, or kidney diseases, hypertension, and other major types of hepatitis were collected through review of medical records and clinical assessment. Any patients who failed these criteria were excluded.

### Sample collection and DNA extraction

Stool samples were collected in sterile stool collection cups and stored at −20 °C overnight. These samples were then aliquoted in 15 mL Falcon tubes and stored at −80 °C within 30 min of sample collection. Then, using the DNeasy^®^ PowerSoil^®^ Pro Kit, DNA was extracted from patient stool samples, aliquoted, and stored at −80 °C. Quality control was performed using the Invitrogen™ Qubit™ dsDNA HS (High Sensitivity) Assay for DNA concentration, and 1% agarose gel electrophoresis was employed to check for DNA integrity.

### Whole-metagenome sequencing

Genomic DNA samples were shipped to BGI Genomics (Beijing, China) for whole metagenome sequencing using their DNBseq sequencing platform. Quality control was performed on samples received by BGI and evaluated for concentration and DNA integrity.

Bioinformatic analysis was then performed on the raw Fastq files obtained. Adapters were filtered by determining if the sequencing read matches 25% or more of the adapter sequence, and if so, the entire read was removed; if not, the adapters are trimmed from the sequence read. Low-quality reads were then scanned for, and sequencing reads that were less than 150 bp were removed. *N* reads were removed if the *N* content in the sequencing read accounts for 0.1% or more of the entire read. Then, to obtain clean reads, low-quality reads were filtered out and removed if the bases in a sequencing read had a quality value of less than 20, which accounts for 40% or more of the entire read. This data processing was performed by SOAPnuke v.1.5.2 software developed by BGI. SOAPnuke software filter parameters were set as follows: -n 0.001 -l 20 -q 0.4 –adaMR 0.25 –adaMis 3 –outQualSys1 –minReadLen 150.

### Metagenomic analysis

High-quality reads were *de novo* assembled using MEGAHIT v1.0.2 software (Li et al., 2015). Contigs shorter than 200 bp were discarded in subsequent analyses. Genes were predicted over the contigs using MetaGeneMark v3.25 (Zhu et al., 2010). Redundant genes were removed with CD-HIT v4.8.1, applying identity and coverage cutoffs of 95 and 90%, respectively (Fu et al., 2012). To construct the gene abundance matrix, Salmon v1.10.3 software was utilized for quantification (Patro et al., 2017). To generate annotation information, the protein sequences of genes were aligned against functional databases (such as BacMet, CARD, KEGG, EggNOG, COG, Swiss-Prot, CAZy, etc.) using DIAMOND v2.1.11 with an *E*-value cutoff of 1e-5 (Buchfink et al., 2015). Taxonomic annotation was assigned based on the Kraken v2.1.4 LCA algorithm (Wood et al., 2019). For database selection, the Unified Human Gastrointestinal Genome (UHGG) v2.0.2 collection was used, as it includes more than 200,000 human gut microbial genomes (Almeida et al., 2021). To create taxonomic and functional abundance profiles, the Bracken (https://github.com/jenniferlu717/Bracken) software v.3.1 was used with the default setting. To develop the taxonomic and functional abundance profiles, the Bracken (https://github.com/jenniferlu717/Bracken) software v.3.1 was used with the default setting (Lu et al., 2017).

### Microbial diversity analysis

Based on the abundance profiles, the features (genera, phyla, and KOs) with significantly differential abundances across groups were determined using Wilcoxon's rank sum test (Matsouaka et al., 2018). Differentially enriched KEGG pathways were identified according to Reporter scores, which is a statistical measure used to evaluate the significance of biological features based on *p*-value and not raw values (Patil and Nielsen, 2005). An absolute value of 1.65 or higher for the reporter score was used as the detection threshold for significance. The alpha diversity was quantified using the Shannon index, Chao1 index, and Simpson index, which were calculated based on the relative abundance profiles at the gene, genus, and KO levels, respectively, with the R package. The beta diversity was calculated using Bray-Curtis distance and Jensen-Shannon Divergence distance (Majtey et al., 2005; Bray and Curtis, 1957). Kruskal-Wallis *H* test assessed phylum-level differences; multivariate ordination used NMDS (Bray-Curtis), PCA, and PCoA with ANOSIM validation. Statistical analysis of the Wilcoxon rank test and Kruskal-Wallis *H* test was calculated using the R project (R Core Team, 2016).

## Results

Metagenomics addresses the microbial population genome as a research object. In this pilot study, the microbial population in samples of five defined groups—normal, post-HCV, treated-HCV, liver disease, and HCV-HCC—was assessed as the main research objective. Then, functional gene sequencing analysis for these microbial populations was categorized into four sections: examining gene overlap and differential abundance, functional annotation and pathway analysis, taxonomic composition and diversity, and statistical and multivariate analysis.

**Figure 1:**
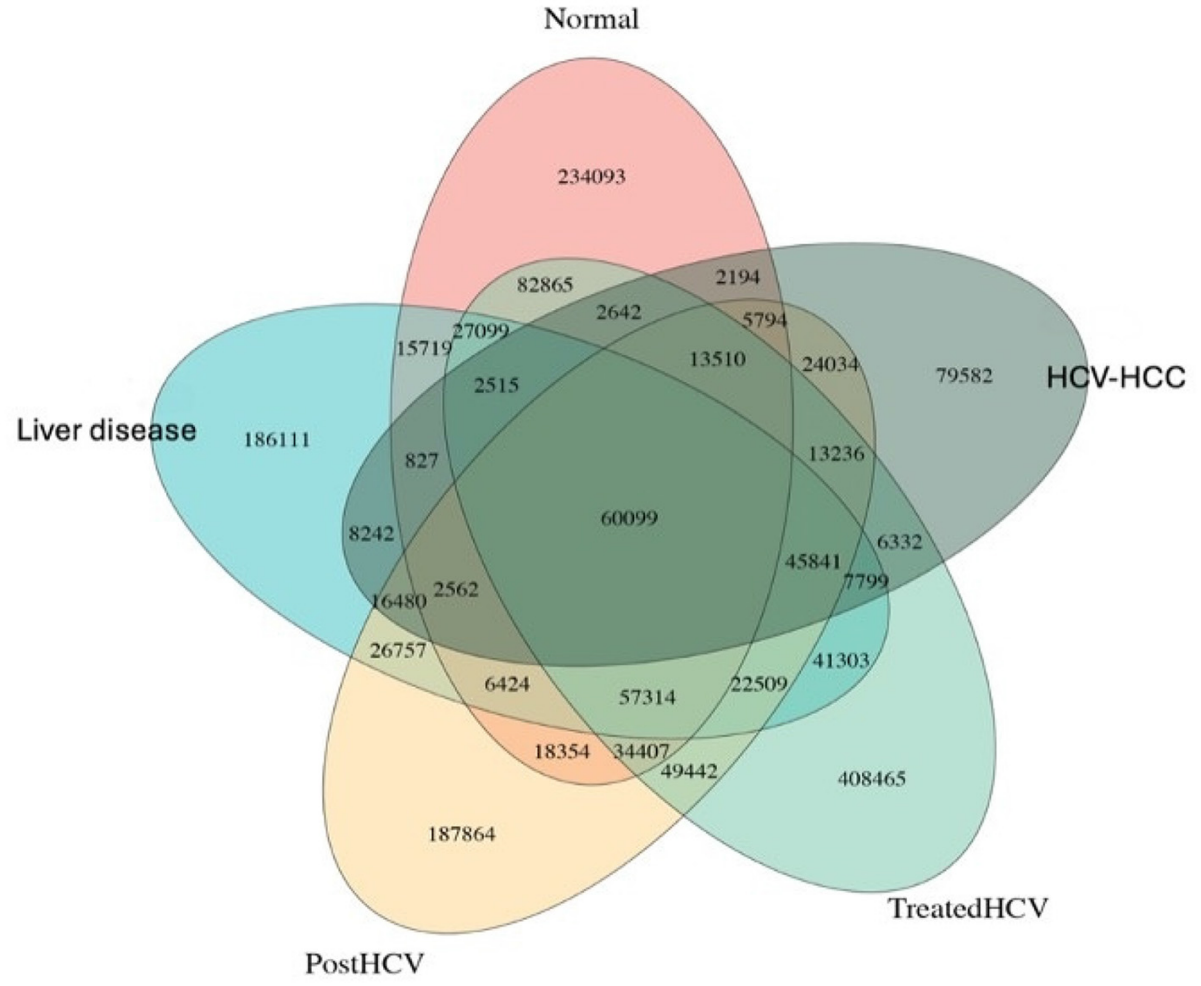
Venn diagram of unique gene sets for stage-specific groups. Venn diagram showing overlap and unique gene sets among normal, liver disease, post-HCV, treated-HCV, and HCV-HCC groups from metagenomic sequencing. This comparative analysis highlights both shared molecular signatures and condition-specific gene expression patterns associated with liver disease progression and hepatitis C virus (HCV)-related hepatocellular carcinoma (HCC).

Venn analysis was performed to identify the number of overlapping genes among different groups, thereby examining the interrelationship of genes. This Venn diagram illustrates the overlap and unique gene sets among the five groups (normal, liver disease, post-HCV, treated HCV, and HCV-HCC), derived from the metagenomic sequencing of microbial gene catalogs. From a genomics perspective, the diagram quantifies shared and distinct microbial genes, reflecting functional conservation or divergence across etiologies. This also provides insight into shared and distinct functional potentials. Significant overlap was observed between the gene content in normal control samples and treated HCV samples. This suggests the recovery of genes encoding beneficial functions in the treated-HCV group after DAA treatment. Also, this substantial gene overlap points toward partial microbiome recovery post-treatment. Gene overlap was also observed between the liver disease and post-HCV groups, which suggests the persistent microbial imbalance or dysbiosis in these groups. However, a unique gene set marked the microbial composition of the HCC group, which may serve as a microbial contribution to oncogenesis (Figure 1).

**Figure 2:**
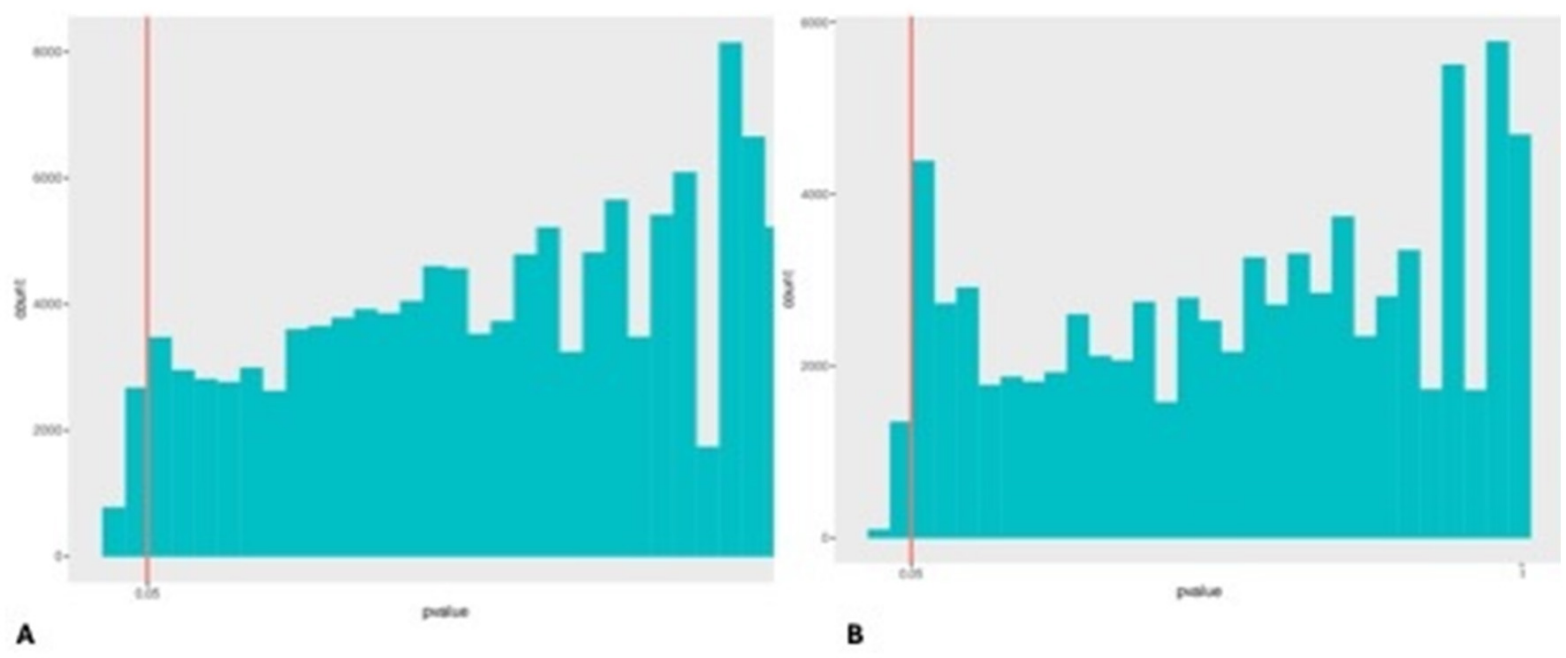
Evaluation of differential abundant genes pairwise amongst groups via *p*-value distribution. Histograms depict the distribution of *p*-values obtained from statistical tests assessing differential significance across two datasets. The red vertical line indicates the conventional significance threshold show a relatively uniform pattern with a modest accumulation of low *p*-values near the significance threshold. **(A)** Distribution for differentially abundant genes between post-HCV and HCV-HCC samples. **(B)** Distribution of *p*-value for differentially abundant genes between post-HCV and treated HCV samples demonstrates a near-uniform pattern but with a slightly higher proportion of tests below the *p* = 0.05 threshold.

As the Venn diagram above illustrates the overlap and unique gene sets among the five groups analyzed, differential gene abundance among the groups was assessed using the non-parametric rank sum test with False Discovery Rate (FDR) correction for multiple comparisons. Barplots of *p*-value distributions revealed statistically significant differences (adjusted *p* < 0.05) in the abundance of specific microbial genes across key group comparisons, highlighting functional shifts (Figure 2).

The application of FDR correction to the rank sum test results ensures a robust identification of differentially abundant genes, controlling for false positives. The pronounced peak of significant differences in Figure 2A (post-HCV vs. HCV-HCC) underscores the substantial genomic and functional divergence associated with HCC development in this cohort.

An unexpected finding was the relatively low number of differentially abundant genes between the post-HCV (untreated clearance) and treated HCV groups (Figure 2B), as a more substantial functional recovery might have been anticipated following Direct-Acting Antivirals (DAA) treatment. This observation suggests that persistent functional dysbiosis might occur even after viral eradication, potentially due to factors like the duration of chronic infection before treatment, incomplete clearance of specific inflammatory microbial taxa, limitations in functional annotation accuracy, or the influence of underlying host factors not fully accounted for in this pilot study.

The significant functional differences observed in comparisons involving the HCV-HCC group highlight a microbiome potentially adapted to support oncogenesis. Conversely, the partial functional recovery noted in the treated HCV group suggests that while DAA therapy is effective against the virus, adjunctive therapeutic interventions targeting the microbiome (e.g., specific probiotics, prebiotics, or potentially bile acid modulators) could be explored to restore microbial functional balance further and potentially mitigate long-term risks.

### Functional annotation and pathway analysis

**Figure 3:**
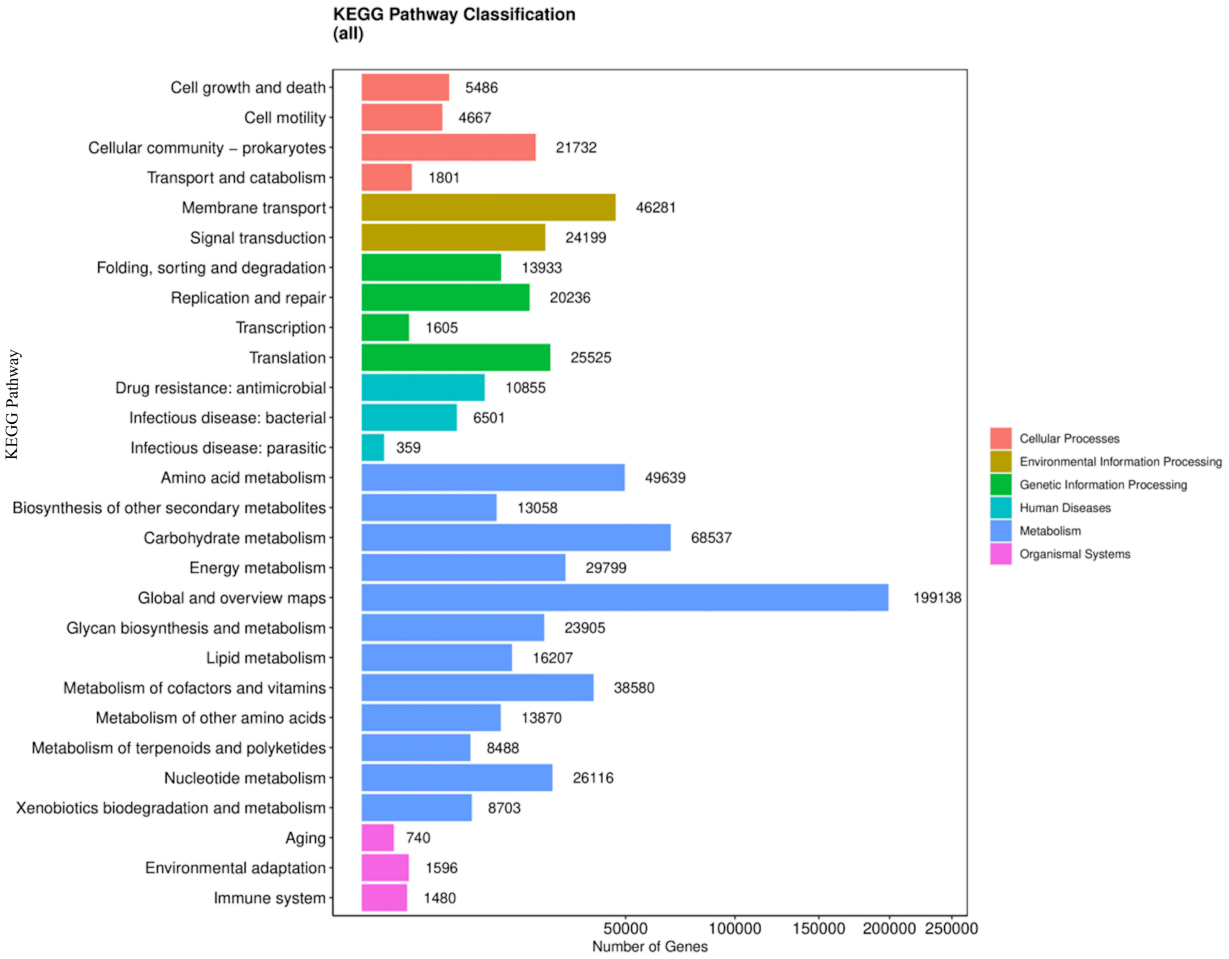
KEGG pathway classification of annotated genes for all groups. Barplot summarizing KEGG orthology (KO) terms across all groups, categorized by function (e.g., metabolism, genetic information processing). Metabolic pathways, particularly Global and overview maps (199,138 genes) and Carbohydrate metabolism (68,537 genes), accounted for the largest proportion of annotated genes, reflecting a strong enrichment in metabolic activities. In contrast, pathways such as Aging (740 genes) and Infectious disease: parasitic (359 genes) were represented by relatively few genes. Overall, the classification highlights that the majority of annotated genes are involved in metabolic and genetic information processing functions.

**Figure 4:**
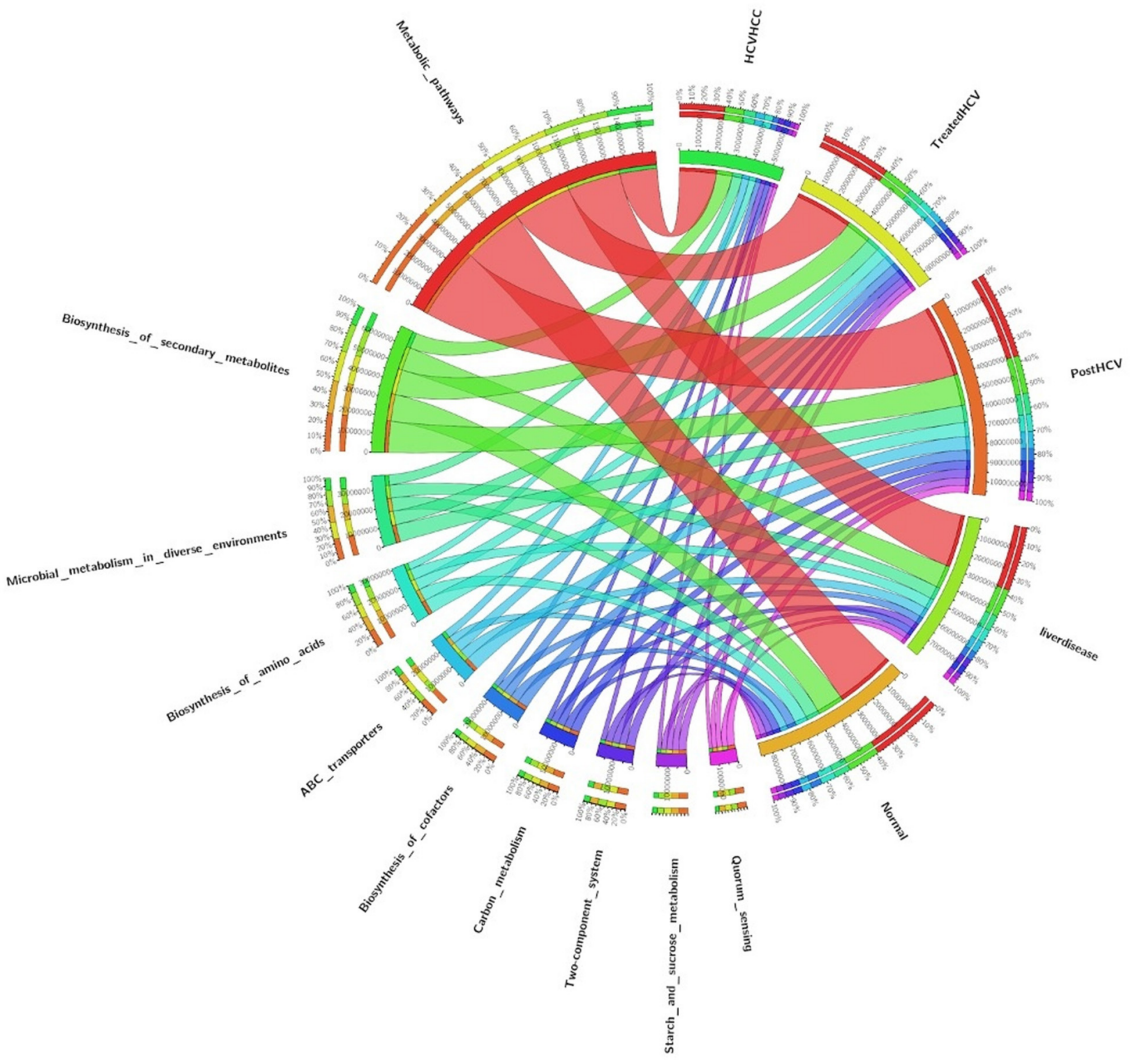
Circos Plot KEGG_level3 of enriched biological pathways in comparison amongst all groups. This figure visualizes the interconnections between metabolic pathways (left half of the circle) and clinical groups (right half), providing a global overview of pathway enrichment patterns across different HCV disease states. Pathways represented include Metabolic pathways, Biosynthesis of secondary metabolites, Microbial metabolism in diverse environments, Biosynthesis of amino acids, ABC transporters, Biosynthesis of cofactors, Carbon metabolism, Two-component system, Starch and sucrose metabolism, Oxidative phosphorylation, and Sulfur metabolism. The disease categories comprise HCV/HCC (HCV with hepatocellular carcinoma), treated HCV, post-HCV, liver disease, and normal controls. his integrative visualization highlights the metabolic reprogramming and dysregulation associated with chronic HCV infection, its treatment, and sequelae, underscoring key biological processes potentially involved in hepatocarcinogenesis and metabolic adaptation in the liver microenvironment.

Metabolism-related KEGG pathways dominated across groups (Figure 3), with carbohydrate and amino acid metabolism comprising the largest shares of annotated genes. Disease states (liver disease, post-HCV, HCV-HCC) showed relative enrichment in inflammatory pathways (e.g., NF-κB signaling) and xenobiotic metabolism, contrasting balanced profiles in normal controls and treated-HCV samples. Circos plots (Figure 4) further revealed bile acid metabolism enrichment in diseased states and LPS biosynthesis/xenobiotic pathways in HCV-HCC, aligning pro-inflammatory functions with dysbiosis severity. However, distinct patterns emerged between groups. Disease states (liver disease, post-HCV, HCV-HCC) showed relative enrichment in genes assigned to inflammatory pathways (e.g., pathways potentially linked to NF-κB signaling, a key regulator of inflammation) and xenobiotic metabolism (involved in processing foreign compounds, including toxins). In contrast, normal controls and DAA-treated HCV samples appeared to maintain more balanced metabolic profiles, suggesting a healthier functional state.

Pathways related to bile acid metabolism were found to be enriched in disease states compared to controls. Microbial modulation of bile acids is significant, as bile acids act as signaling molecules influencing both microbial composition and host immune responses via the gut-liver axis. Circos plots (Figure 4) provided a visualization of the top enriched KEGG Level 3 pathways for all groups. As shown below in Liver Disease there was a marked enrichment in inflammatory pathways (including bile acid metabolism and potentially NF-κB signaling pathways). A significant functional shift was evident, with the HCV-HCC group showing marked enrichment in pathways related to xenobiotic metabolism, oxidative stress response, and LPS production. This functional profile is consistent with microbial contributions to inflammation, DNA damage, and immune evasion, all processes implicated in oncogenesis. There is partial restoration of beneficial pathways (e.g., potentially related to SCFA production) in the DAA-treated HCV group although evidence of persistent dysbiosis remained (e.g., presence of resistance genes). The HCV-HCC group was significantly enriched in pathogenesis-related pathways (e.g., potentially including toxin degradation or virulence factor pathways) compared to the partially recovered profile of the treated HCV group.

The observed enrichment of microbial bile acid metabolism pathways in diseased states suggests a potentially important role for the gut microbiota in modulating host bile acid pools and signaling via the gut-liver axis in this Egyptian cohort. Prior studies focusing on different populations or etiologies may not have emphasized this aspect, enrichment of bile acid metabolism could represent a therapeutic target. The apparent functional shift toward inflammatory and potentially oncogenic pathways in the HCV-HCC group underscores the plausible contribution of the gut microbiome to disease progression in these patients. Conversely, the partial functional restoration seen in the treated HCV group supports the efficacy of antiviral therapies in mitigating some, but perhaps not all, aspects of microbial dysbiosis.

**Figure 5:**
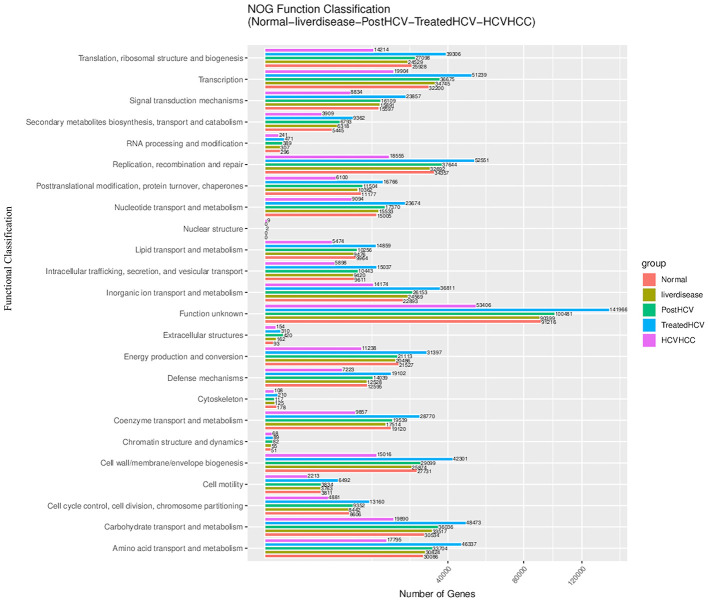
eggNog functional classification of gut microbiome genes across different liver disease stages in Egyptian patients. This bar plot visually represents the distribution of predicted microbial gene functions across the five study groups. Distinct shifts in functional gene abundance are observed between disease stages, there are higher representation of genes involved in energy production, amino acid metabolism, and membrane biogenesis in HCVHCC and treated HCV groups which suggest microbial adaptation to host disease progression and antiviral treatment.

**Figure 6:**
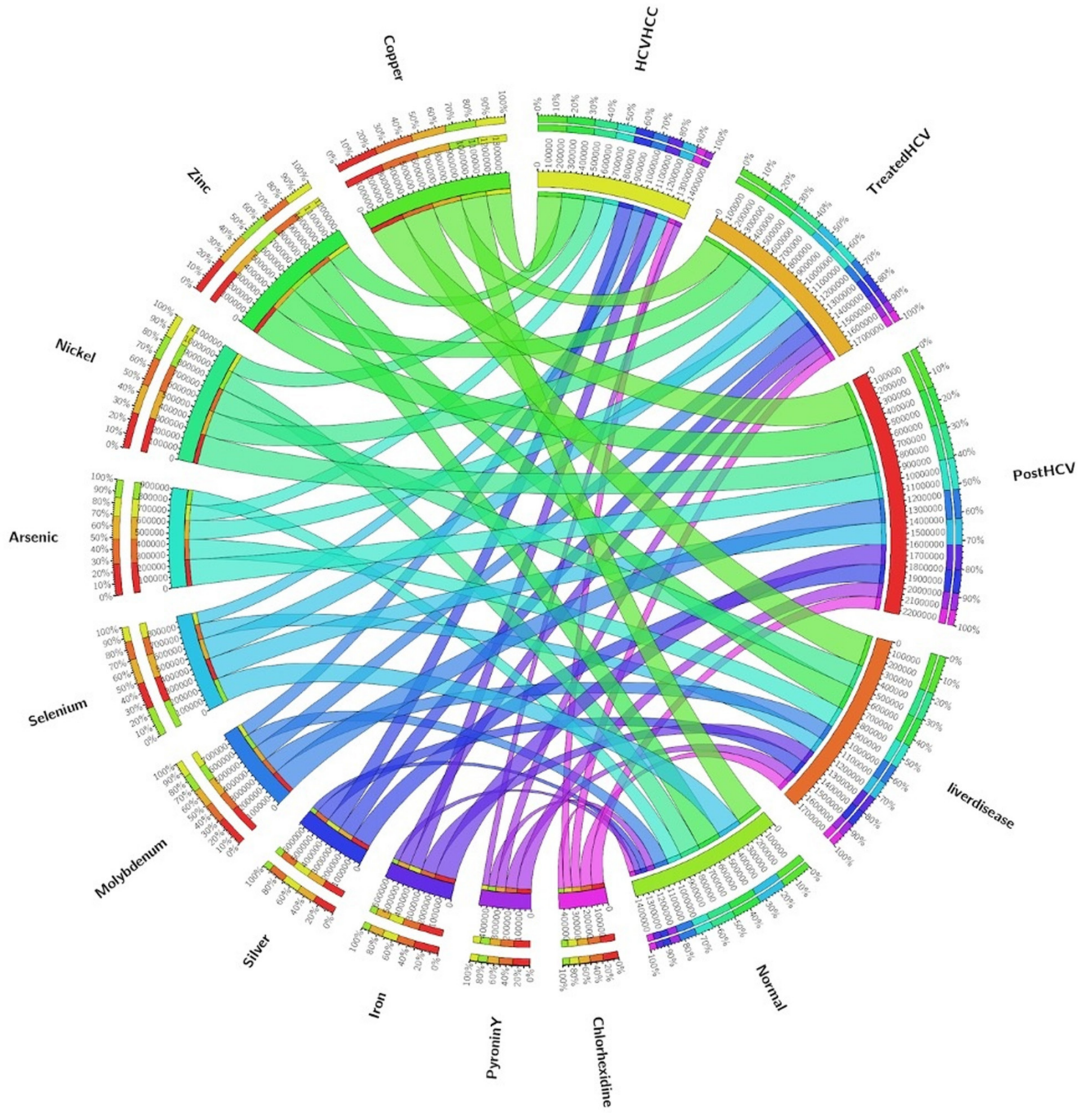
Circos Plot BacMet of resistant genes between groups. In this Circos plot complex associations between various trace metals and disease states. The left half of the circle represents individual elements/metals, including Copper, Zinc, Nickel, Arsenic, Selenium, Molybdenum, Silver, and Iron, as well as certain chemical compounds (Pyronin Y and Chloranilidine). The right half represents distinct clinical groups, namely HCV/HCC (hepatitis C virus with hepatocellular carcinoma), treated HCV, post-HCV, Liver disease, and Normal controls. This visually represents how metal metabolism and trace element homeostasis differ across the HCV disease spectrum, revealing potential biomarkers and metabolic alterations associated with liver pathophysiology.

**Figure 7:**
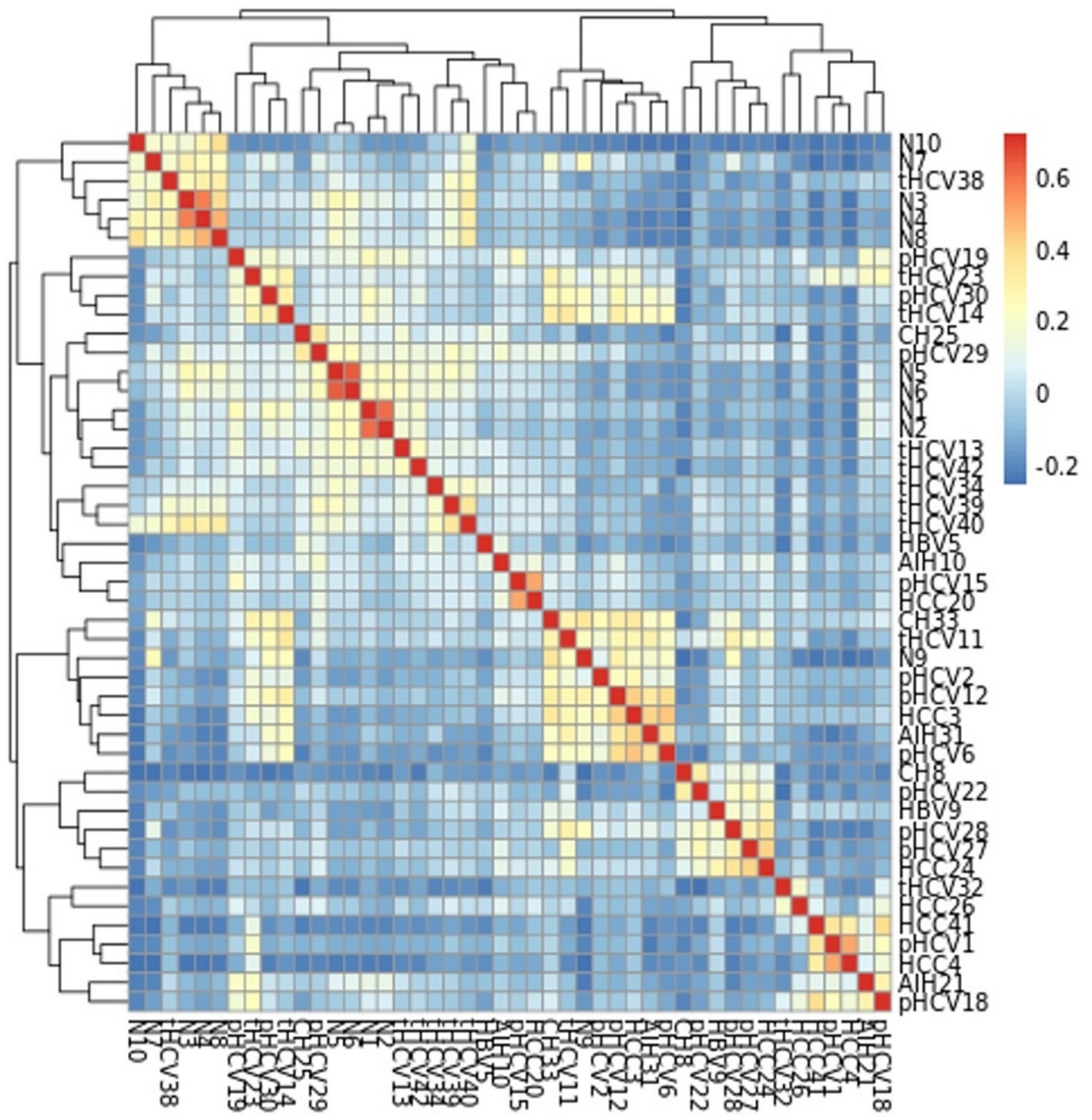
Heatmap Bray-Curtis taxonomic abundance of all samples. This hierarchical clustering heatmap shows pairwise correlations among gut microbiome profiles across samples from Egyptian patients with various liver disease stages and HCV-HCC. The color intensity represents correlation strength with warmer colors (red to yellow) indicating a strong positive correlation and cooler colors (blue) representing negative to weak correlation. Clustering across both axes reveal distinct microbial community patterns, suggesting that gut microbiome composition varies with liver disease progression and HCV infection.

**Figure 8:**
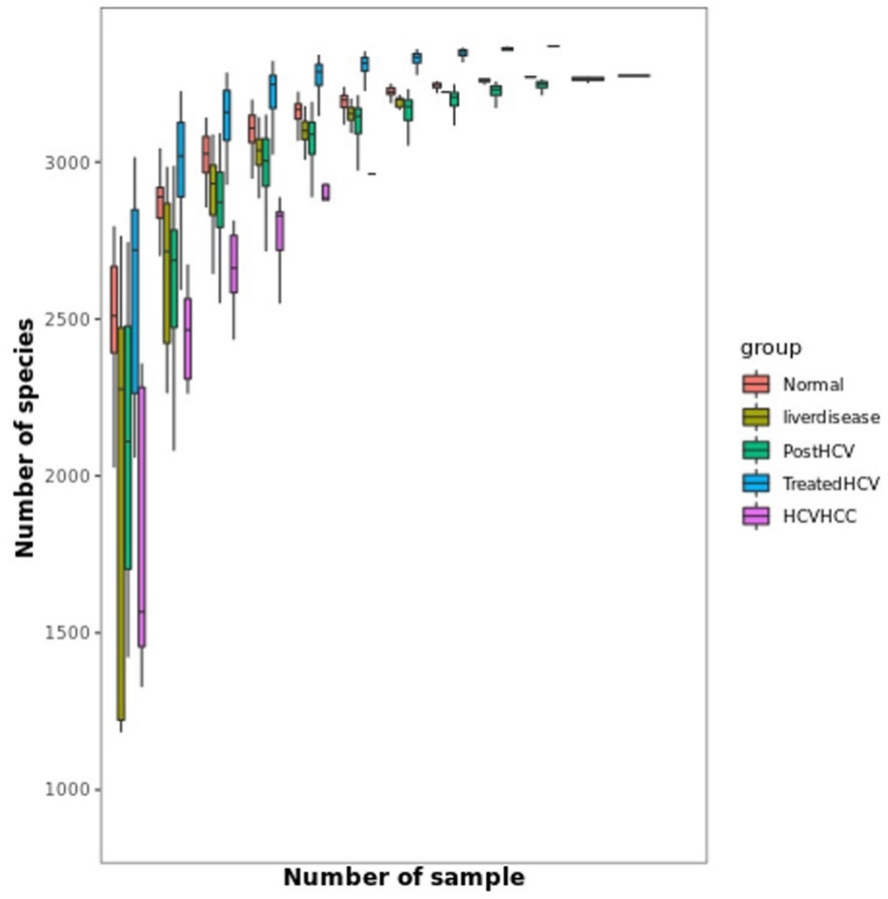
Rarefaction curve for comparison of species richness across study groups. Rarefaction analysis illustrates the relationship between sequencing depth (number of samples) and observed species richness (number of species) across different groups: normal, liver disease, post-HCV, treated HCV, and HCV-HCC. Each curve represents the average accumulation of detected species as sampling effort increases, with shaded or boxed regions indicating variability among replicates. The curves approach a plateau, suggesting adequate sequencing coverage for most groups. Notably, treated HCV and post-HCV samples display slightly higher species richness compared to HCV-HCC and Liver disease groups, indicating potential alterations in microbial diversity associated with disease progression and antiviral treatment.

Functional annotation using the EggNOG database (v5.0) provided complementary insights into gene functions (Figures 5–8). Similar to KEGG, metabolism-related functions dominated, but EggNOG allowed finer-grained analysis of functional categories. Comparisons revealed enrichment of functions related to replication, recombination, and repair in diseased states, potentially reflecting microbial stress responses. Signal transduction mechanisms were also prominent, particularly in HCV-HCC, suggesting altered microbial communication and host interaction. Treated HCV samples showed partial normalization of these functional profiles compared to post-HCV.

The figure above provides a high-level view of microbial functional diversity, with OGs related to energy production, cell wall biogenesis, or stress response. The trend suggests that diseased states (liver disease, post-HCV, HCV-HCC) are enriched in stress response or resistance-related OGs, while normal and treated HCV samples have balanced metabolic OGs. The enrichment of stress response OGs in diseased states correlates with liver inflammation and fibrosis, suggesting that targeting these functions (e.g., with antibiotics) could mitigate disease severity.

The BacMet database (v2.0) specifically identified genes potentially conferring resistance to biocides and metals, as well as potential antibiotic resistance genes (ARGs; Figure 6). Notably, an enrichment of various resistance genes was observed in diseased states, particularly in post-HCV and HCV-HCC groups. The persistence of these genes, potentially including ARGs, even in the treated HCV group (Figure 6), was an important finding. This suggests that while DAA treatment clears the virus, it may not fully resolve the reservoir of resistance genes within the gut microbiota. The source could be horizontal gene transfer among bacteria or the selection pressure from past antibiotic exposures (common in clinical settings). The persistence of ARGs is a public health concern and indicates that the gut microbiome in these patients might remain a reservoir for resistance determinants. The enrichment in HCV-HCC might also reflect adaptation to a hostile gut environment characterized by inflammation and altered metabolite profiles.

### Taxonomic composition and diversity

Consistent with functional findings, normal and treated HCV samples were relatively enriched in taxa often considered beneficial, such as members of the *Bifidobacterium* genus and the Firmicutes phylum, known SCFA producers. In contrast, the HCV-HCC group showed increased relative abundance of potentially pathogenic or pro-inflammatory taxa, including members of the *Enterobacteriaceae* family and the *Proteobacteria phylum*, indicative of significant dysbiosis. Bray-Curtis distance-based heatmaps (Figure 7) confirmed taxonomic similarities based on overall community structure, visually demonstrating that normal and treated HCV samples clustered more closely together, while the HCV-HCC group formed a distinct cluster.

The clustering of treated HCV with normal samples suggests a degree of taxonomic recovery following DAA treatment, reinforcing its efficacy in partially restoring the microbiome structure. The distinct dysbiotic taxonomic profile observed in HCV-HCC correlates with findings of increased inflammation and oncogenic functional potential. This suggests that specific taxa (e.g., Escherichia) could serve as biomarkers and/or therapeutic targets. Interventions aimed at increasing Firmicutes (e.g., via probiotics or prebiotics) could improve outcomes, although this requires clinical validation.

**Figure 9:**
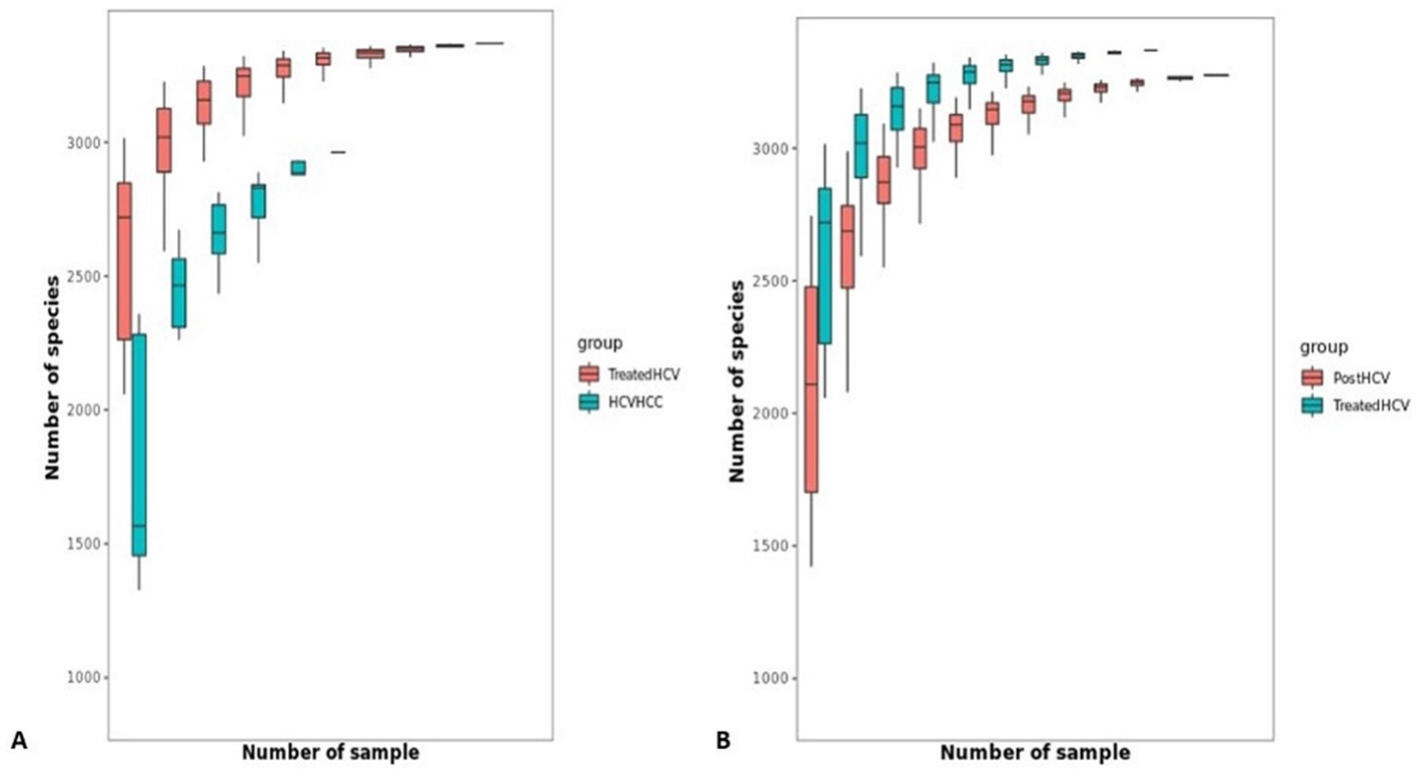
Rarefaction curve for pairwise comparison of species richness between groups. **(A)** Comparison of species richness between treated HCV and HCV-HCC groups. The rarefaction curves show that the treated HCV group (red) exhibits higher microbial diversity across increasing sequencing depths compared to the HCV-HCC group (teal), indicating a reduction in species richness associated with hepatocellular carcinoma (HCC) development. **(B)** Comparison between post-HCV and treated HCV groups. Both groups display similar rarefaction trends, with the treated HCV samples showing a slightly higher plateau, suggesting that antiviral treatment may partially restore microbial diversity after HCV clearance. In both panels, the *x*-axis represents the number of samples (sequencing depth), and the *y*-axis shows the number of observed species. The boxplots illustrate variation in species counts across samples within each group.

To assess microbial richness and how species diversity changes with sample size, rarefaction curves were employed. Rarefaction curves (Figures 8, 9) show a visual representation of how species diversity changes with sample size and indicate if additional sampling may yield different results. Rarefaction curves for normal samples exhibited the highest richness, while HCV-HCC showed the lowest, reflecting severe dysbiosis. Treated HCV samples showed improved richness compared to post-HCV, indicating partial recovery. Rarefaction curves plateaued across groups (Figures 8, 9), confirming adequate sequencing depth (~10–20 million clean reads/sample post-QC; median Phred >30), with ANOVA-confirmed richness differences (*p* < 0.05). The reduced diversity in HCV-HCC underscores a loss of microbial functionality, while improved diversity in treated HCV supports microbiome restoration.

### Differentially abundant taxa

**Figure 10:**
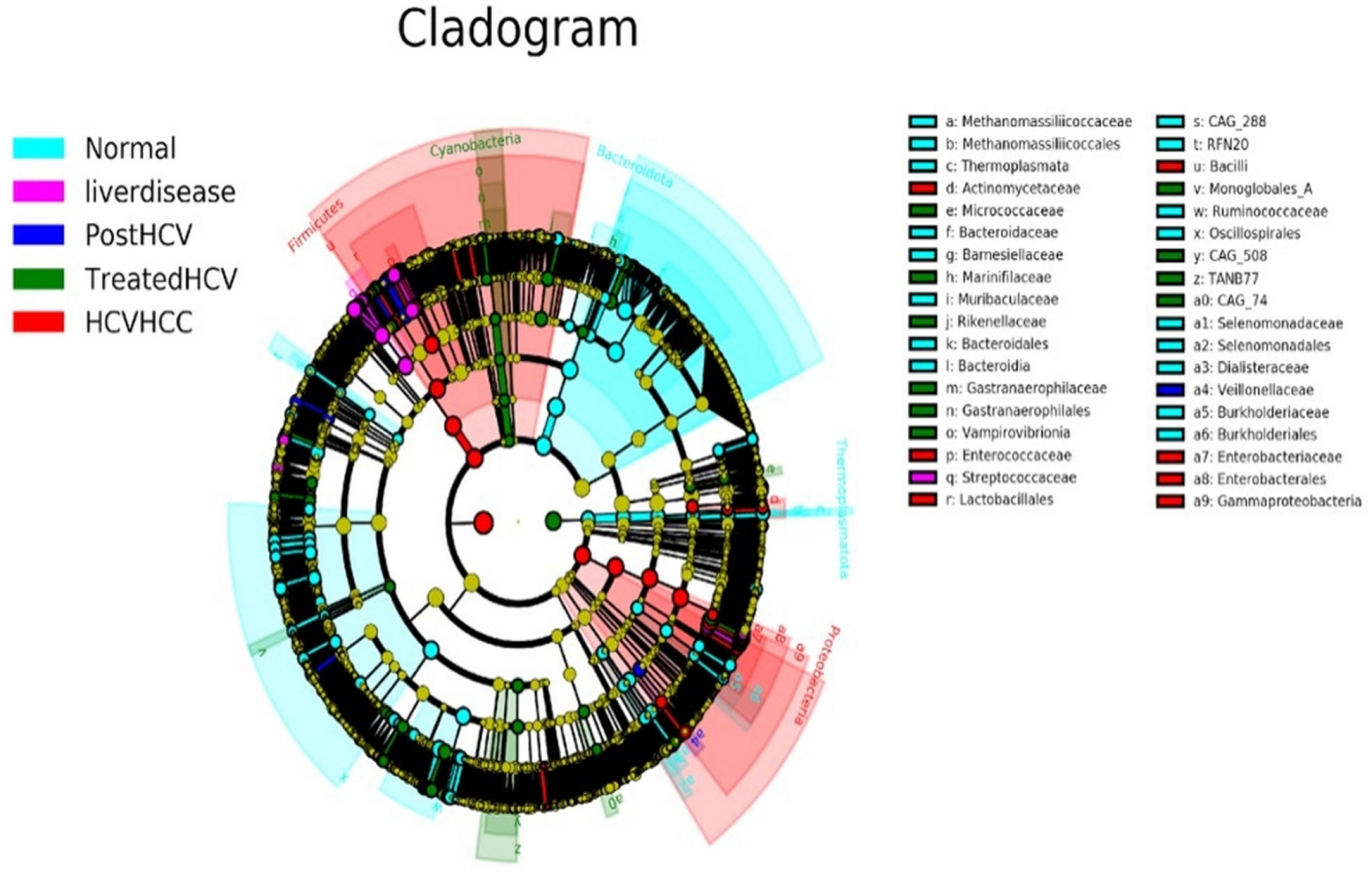
LEfSe-generated cladogram representing the differential microbial taxa among all groups at the phylum level. The cladogram displays the taxonomic representation of significantly different bacterial clades among the five groups: normal, liver disease, post-HCV, treated HCV, and HCV-associated hepatocellular carcinoma (HCV-HCC). The circles represent taxa, with the diameter proportional to their relative abundance. The taxonomic levels are arranged from phylum (innermost circles) to genus (outermost circles). Colored sectors indicate the most abundant taxa within each group as determined by LEfSe analysis. Taxa enriched in each group are highlighted in the corresponding color, while yellow nodes indicate taxa with no significant differential abundance. The right legend lists the taxa corresponding to the labeled nodes (a–a9) in the cladogram.

Linear discriminant analysis Effect Size (LefSe) analysis was used to identify specific taxa that were differentially abundant between groups, visualized using cladograms (Figure 10). The LEfSe-generated cladogram highlights differentially abundant taxa, with branches showing phylogenetic relationships. This analysis highlighted key taxonomic shifts associated with the disease state. For example, members of the genus *Escherichia* (within the *Proteobacteria phylum*) were found to be significantly enriched in the HCV-HCC group, consistent with its potential role as a pathobiont in this context. Conversely, taxa often considered beneficial, such as *Lactobacillus*, were found to be enriched in the treated HCV group compared to other diseased states, reflecting a potential marker of recovery. These differentially abundant taxa could serve as potential biomarkers for disease staging, monitoring treatment response, or identifying therapeutic targets. For instance, the enrichment of *Lactobacillus* in treated HCV patients might suggest that supplementation with specific *Lactobacillus* strains could be explored as an adjunctive therapy to support microbiome restoration further, although clinical trials would be necessary to confirm efficacy.

### Statistical and multivariate analyses

**Figure 11:**
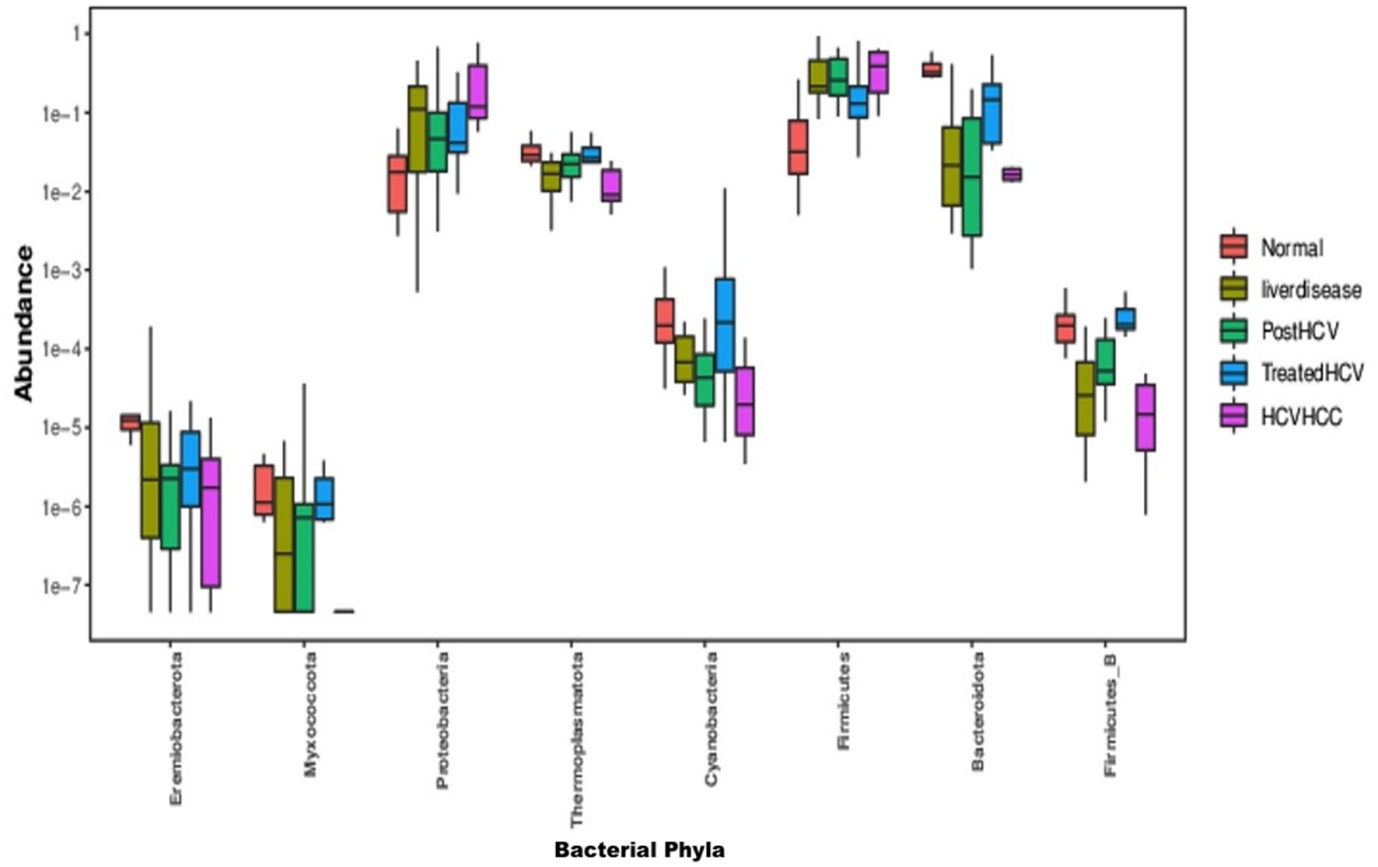
Kruskal-Wallis test visualizing relative abundance of dominant bacterial phyla across all groups. Boxplots display the distribution of relative abundances of the major bacterial phyla identified in the gut microbiota of the five study groups: normal (red), liver disease, post-HCV, treated HCV, and HCV-associated hepatocellular carcinoma (HCV-HCC). Each box represents the interquartile range (IQR) with the median indicated by the central line, and whiskers denote the variability outside the upper and lower quartiles. Variations in phylum-level composition demonstrate distinct microbial community structures among disease states and treatment outcomes.

The Kruskal-Wallis test confirmed statistically significant differences (*p* < 0.05) in phylum-level abundances across groups (Figure 11), with Proteobacteria significantly enriched in HCV-HCC and Firmicutes in normal controls. NMDS, PCA, and PCoA ordination consistently separated groups (ANOSIM *p* < 0.05), with HCV-HCC forming the most distinct cluster and treated-HCV grouping closely with controls. The test identified statistically significant differences (*p* < 0.05) at the phylum level. Specifically, it confirmed the significant enrichment of Proteobacteria in the HCV-HCC group and the relative enrichment of Firmicutes in the normal control samples compared to the diseased groups. These findings provide statistical support for the observed phylum-level dysbiosis patterns. Such broad taxonomic shifts may serve as accessible biomarkers for diagnosing or monitoring liver disease progression, although genus- or species-level markers often offer greater specificity.

**Figure 12:**
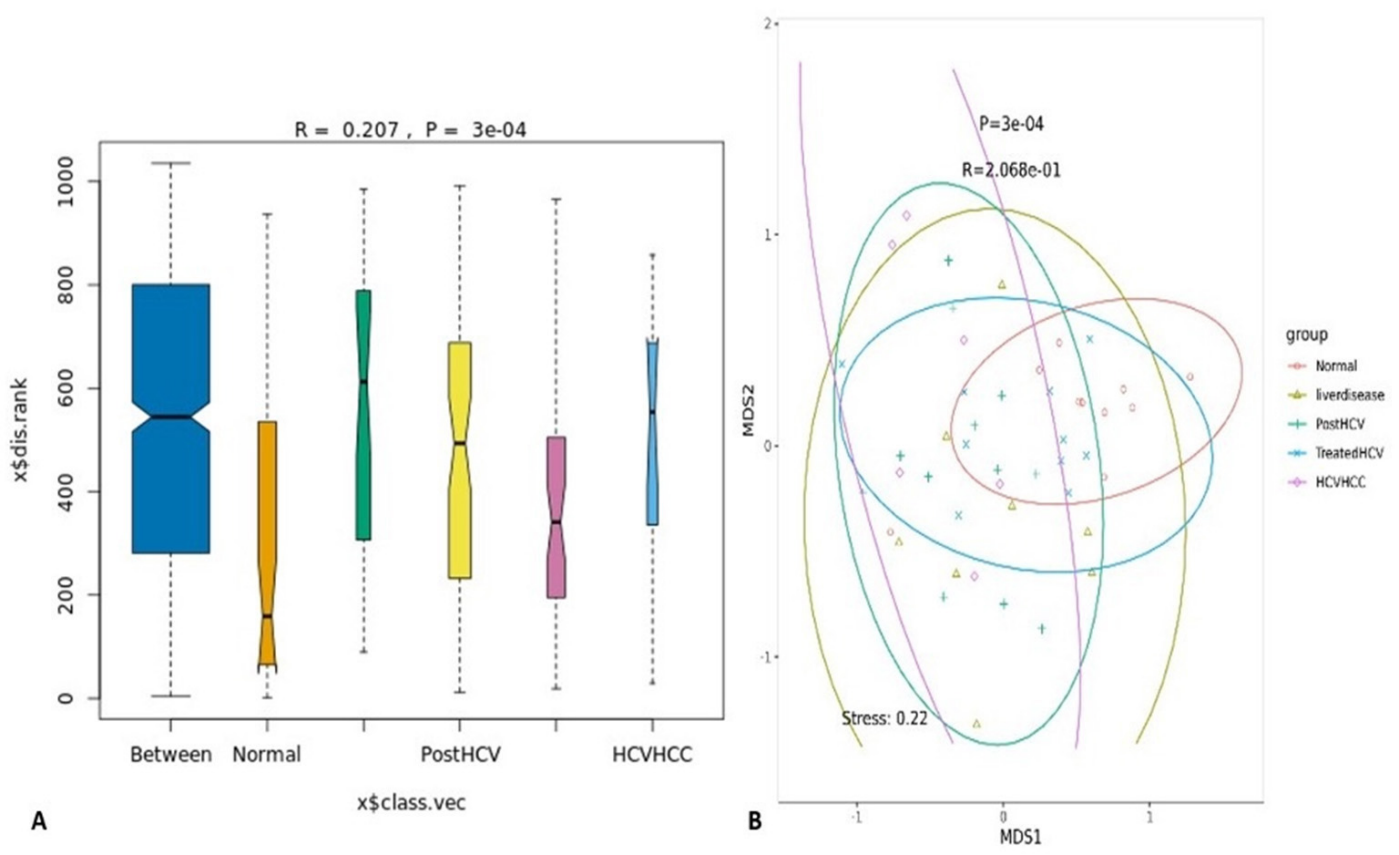
Beta diversity analysis of gut microbiota among study groups. **(A)** Boxplot illustrating pairwise distances (Bray–Curtis dissimilarity) between and within the five study groups: normal, liver disease, post-HCV, treated HCV, and HCV-associated hepatocellular carcinoma (HCV-HCC). Differences in microbial community structure were assessed by PERMANOVA (*R* = 0.207, *p* = 3 × 10^−^4). **(B)** Non-metric multidimensional scaling (NMDS) ordination plot based on Bray–Curtis distances showing the clustering of samples according to group. Each point represents an individual sample, and the colored ellipses indicate 95% confidence intervals for each group. The NMDS stress value (0.22) reflects an acceptable goodness of fit for the ordination. Distinct group clustering patterns suggest significant compositional differences in gut microbiota among disease stages and treatment conditions.

**Figure 13:**
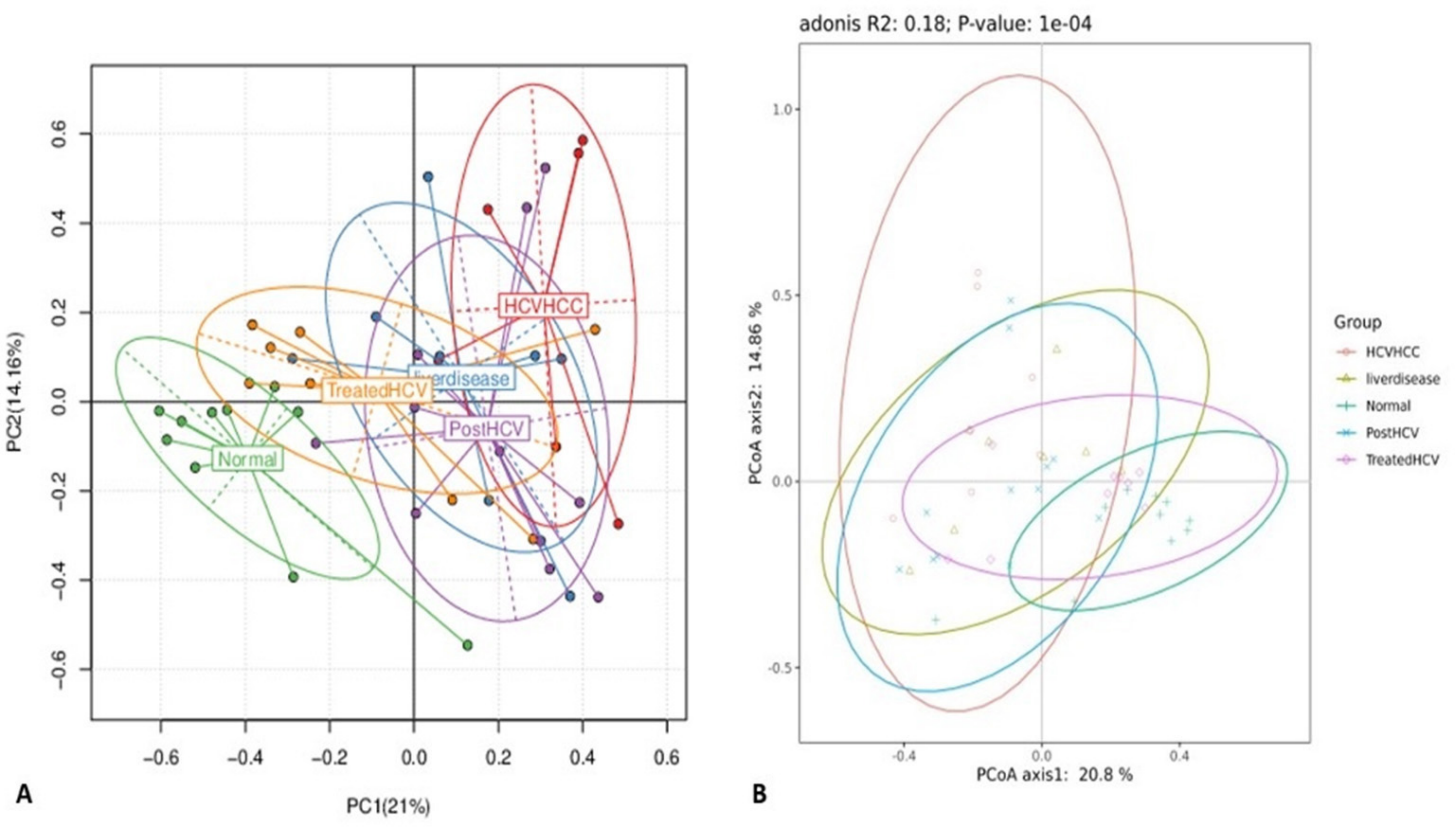
Principal coordinate analysis (PCoA) and principal component analysis (PCA) of gut microbial community composition among study groups. **(A)** Principal component analysis (PCA) plot based on relative abundance profiles illustrating the separation of microbial communities across the five study groups: normal, liver disease, post-HCV, treated HCV, and HCV-associated hepatocellular carcinoma (HCV-HCC). Each ellipse represents the 95% confidence interval for each group, indicating distinct clustering patterns along PC1 (21%) and PC2 (14.16%). **(B)** Principal coordinate analysis (PCoA) based on Bray–Curtis distances showing the overall beta diversity among the same groups. The adonis test confirmed significant differences in microbial composition between groups (*R*^2^ = 0.18, *p* = 1 × 10^−^4). The separation of samples along the first two axes (20.8 and 14.86% of explained variance) reflects disease-associated microbial shifts.

Following statistical validation, multivariate ordination techniques, including Non-Metric Multidimensional Scaling (NMDS, Figure 12), Principal Component Analysis (PCA, Figure 13A), and Principal Coordinates Analysis (PCoA, Figure 13B), were employed to visualize overall microbial community differences (beta diversity) between the groups based on genus-level taxonomic profiles. All three methods consistently showed significant separation between the groups. NMDS coupled with ANOSIM (Analysis of Similarities) confirmed statistically significant differences in community composition and dispersion between groups (*p* < 0.05), with the HCV-HCC group often showing the most distinct clustering and dispersion pattern.

Similarly, permutation-based statistical tests applied to PCA and PCoA results confirmed substantial community differences. These analyses visually emphasize the distinct microbial profile linked with HCV-HCC, clearly separating it from the normal control and treated HCV groups, which tend to cluster more closely together. An intriguing and unexpected observation was the relatively tight grouping of the treated HCV group with the normal controls in these ordination plots (PCA/PCoA), which indicates substantial taxonomic recovery at the community level.

This contrasts with the functional analyses, which revealed persistent functional dysbiosis. This discrepancy suggests that taxonomic recovery (restoring community structure) may occur before or more easily than full functional recovery after DAA treatment. Possible explanations include the persistence of low-abundance yet functionally important taxa, limits in the depth of functional annotation, or long-term epigenetic changes in the host affecting the gut environment even after taxonomic shifts. This nuanced finding has implications for defining therapeutic endpoints, implying that monitoring functional recovery could be as crucial as tracking taxonomic changes. The distinct microbial profile of HCV-HCC reinforces its potential as a diagnostic marker, while the clustering pattern provides further evidence of partial, mainly taxonomic, microbiome restoration following DAA treatment.

In summary, the results of this pilot study demonstrate a clear pattern of progressive microbial dysbiosis, an imbalance in the gut microbial community structure and function associated with the progression from liver disease to HCV-HCC in the studied Egyptian patients (Table 1 below). Statistically significant functional and taxonomic shifts were identified between disease stages. Patients successfully treated with DAAs showed evidence of partial microbiome recovery, characterized by restored levels of some beneficial taxa (e.g., *Lactobacillus, Bifidobacterium*) and functions (e.g., SCFA production pathways), and an overall community structure closer to that of healthy controls. However, the persistence of certain dysbiotic features suggests that DAA therapy alone may not fully normalize the gut microbiome. The unique microbial signature of HCV-HCC, significantly enriched in potentially pathogenic genes (e.g., LPS biosynthesis, xenobiotic metabolism) and taxa (e.g., Escherichia, Proteobacteria), highlights its potential role in contributing to oncogenesis and its utility for developing diagnostic or prognostic biomarkers.

**Table 1:** Microbial profiles across groups.

Group	Key taxa	Functional profile	Novel findings
Normal	*Faecalibacterium, Bifidobacterium*	SCFA biosynthesis, metabolism-related	*Faecalibacterium* dominance, fiber diet link
Liver Disease	Increased Proteobacteria	Inflammatory pathways, bile acid metabolism	Persistent dysbiosis, resistance genes
Post-HCV	*Escherichia*, Proteobacteria	Inflammatory, resistance genes	Antibiotic use impact, partial recovery
Treated HCV	Restored *Lactobacillus, Bifidobacterium*	Partial SCFA restoration, acetate genes	Strong recovery, DAA efficacy
HCV-HCC	Dominant *Escherichia*, Proteobacteria	LPS, xenobiotic metabolism, cytochrome P450	Cytochrome P450 as HCC biomarker

## Discussion

The gut microbiome profoundly influences liver health through complex bidirectional communication along the gut-liver axis. This interplay involves microbial metabolites, immune signaling, and regulation of intestinal barrier function, collectively modulating host inflammation, metabolism, and potentially oncogenesis (Bray and Curtis, 1957). Dysbiosis, broadly defined as detrimental alterations in microbial composition and function, has been increasingly linked to the progression of chronic liver diseases (Albillos et al., 2020). This pilot study investigates the role of the gut microbiome in normal controls and Egyptian patients with liver disease, post-hepatitis C virus (HCV) clearance without DAA treatment, DAA-treated HCV, and HCV-associated hepatocellular carcinoma (HCC) using metagenomic sequencing. The study characterizes microbial composition and function, identifies stage-specific biomarkers, and explores therapeutic targets in this population with a high prevalence of HCV-related liver disease.

### Distinct gut microflora across liver disease stages

Metagenomic analyses revealed distinct, etiology-specific microbial profiles, reflecting a gradient of increasing dysbiosis from the healthy state to HCV-associated HCC. Samples from normal controls exhibited the highest microbial richness (alpha diversity, Figure 8) and were relatively dominated by members of the Firmicutes phylum, including potentially beneficial genera such as *Faecalibacterium*. *Faecalibacterium* species, particularly *F. prausnitzii*, are well-known producers of the SCFA butyrate, which serves as a primary energy source for colonocytes, strengthens the gut barrier, and possesses potent anti-inflammatory properties by inhibiting NF-κB signaling and promoting regulatory T cell function (Miquel et al., 2013). Liver disease samples showed signs of moderate dysbiosis, characterized by reduced microbial richness compared to controls (Figure 8) and an increased relative abundance of Proteobacteria (including members of the *Enterobacteriaceae* family). This phylum includes many Gram-negative bacteria capable of producing LPS, which can translocate across a compromised gut barrier, reach the liver via the portal vein, and trigger inflammatory responses through Toll-like receptor 4 (TLR4) signaling, contributing to liver injury (Compare et al., 2012). Post-HCV (untreated clearance) displayed persistent dysbiosis, often enriched in genera like Escherichia and depleted in beneficial taxa compared to controls, along with reduced richness (Figure 8). This suggests that spontaneous viral clearance may not be sufficient to restore microbiome homeostasis fully.

Samples from HCV patients treated with (DAA) indicated partial recovery and showed restored relative abundance of specific beneficial taxa like *Lactobacillus* and *Bifidobacterium* and improved microbial richness compared to untreated post-HCV patients (Figure 9B). Furthermore, their overall community structure clustered more closely with normal controls in multivariate analyses (Figures 12, 13), suggesting significant taxonomic restoration. While HCV-HCC samples were characterized by marked dysbiosis, exhibiting the lowest microbial richness (Figure 9A) and a considerable dominance of potentially pathogenic taxa like Escherichia and other Proteobacteria (Figure 10). This profile suggests a pro-inflammatory and potentially oncogenic microbiome composition.

These results align with global studies linking Firmicutes depletion and Proteobacteria enrichment to liver disease progression (Qin et al., 2014). In a study by (Qin et al. 2014), similar Proteobacteria dominance in cirrhosis was reported, supporting the dysbiosis observed in liver disease and post-HCV (Qin et al., 2014). However, the pronounced *Bifidobacterium* restoration in treated HCV contradicts some studies (e.g., Bajaj et al., 2018), which found limited taxonomic recovery post-HCV treatment, possibly due to differences in treatment regimens or regional diets (Bajaj et al., 2018). It is of particular interest, the severe dysbiosis observed in HCV-HCC, by Escherichia dominance. Support for these findings comes from a study by (Ren et al. 2019), who identified Escherichia as an HCC biomarker (Ren et al., 2019).

The study's identification of *Faecalibacterium* as a dominant taxon in normal Egyptian samples is novel, potentially reflecting Egypt's carbohydrate heavy diets, which differ from Western cohorts where Bacteroides often predominates (Yatsunenko et al., 2012). The tight clustering of treated HCV with normal samples in multivariate analyses (Figure 11) is a novel finding, suggesting a stronger taxonomic recovery than previously reported, possibly due to Egypt's national campaign for treatment of HCV using direct-acting antivirals (DAAs; El Kassas et al., 2018).

These distinct microbial profiles highlight the microbiome's role in disease staging, with Escherichia in HCV-HCC as a potential diagnostic marker and *Bifidobacterium* restoration in treated HCV indicating therapeutic success (Ponziani et al., 2019; Loomba et al., 2017).

We hypothesize that dietary fiber drives SCFA production, however, future studies should incorporate food frequency questionnaires to quantify dietary influences, aligning with global recommendations (Singh et al., 2017). While Proteobacteria enrichment in HCV-HCC suggests a pro-inflammatory role, our cross-sectional design cannot distinguish whether dysbiosis drives HCC or results from it. Mouse models demonstrating reduced HCC incidence post-antibiotic treatment support a causal link (Ma et al., 2018), but human studies are needed. We propose longitudinal studies and fecal microbiota transplantation (FMT) trials to test causality, building on preclinical evidence (Dapito et al., 2012).

### Molecular links between liver disease stages

Functional annotations using KEGG and EggNOG databases provided insights into the shifting molecular capabilities of the gut microbiome across the different patient groups (Figures 3–6). Normal controls and treated HCV samples showed relative enrichment in genes associated with SCFA biosynthesis and potentially linked to butyrate production by *Faecalibacterium* and acetate production by *Bifidobacterium*. Thus, supporting metabolic homeostasis and anti-inflammatory effects. In contrast, liver disease and post-HCV samples exhibited overlapping gene enrichments related to pro-inflammatory pathways by potentially involving NF-κB signaling and altered bile acid metabolism, reflecting persistent functional dysbiosis (Figure 4). Differential gene abundance analyses (adjusted *p* < 0.05) indicated a partial restoration of SCFA production pathway genes in treated HCV compared to post-HCV, yet also revealed the persistence of potentially detrimental genes, including antibiotic resistance genes (ARGs) identified via BacMet annotation (Figures 4, 6). This highlights the complexity of functional recovery, suggesting that eliminating the virus doesn't necessarily resolve all aspects of functional dysbiosis.

HCV-HCC samples displayed a major functional shift, characterized by significant enrichment (adjusted *p* < 0.05) in genes involved in LPS biosynthesis, xenobiotic metabolism (including microbial cytochrome P450 homologs), oxidative stress response, and potentially toxin production/degradation (Figure 4). This functional profile suggests a microbiome actively contributing to a pro-inflammatory, mutagenic, and immune-evasive microenvironment conducive to cancer development and progression (Yu and Schwabe, 2017). The enrichment in xenobiotic metabolism pathways is particularly relevant in contexts with potential environmental toxin exposure.

The persistence of resistance genes in post-HCV samples supports findings of (Talaat et al. 2016), highlighting antibiotic resistance as a barrier to microbial recovery in Egypt (Syed and Yadav, 2012). This could impair long-term liver recovery, warranting antibiotic stewardship programs and microbial interventions (e.g., probiotics) to restore eubiosis (El-Sokkary et al., 2021).

However, the strong restoration of SCFA genes in treated HCV contrasts with (Ponziani et al. 2019), who reported limited functional recovery post-DAA therapy, possibly due to Egypt's unique treatment protocols (Ponziani et al., 2019). This is due to Egypt's comprehensive national strategy that combines wide access to locally produced generic direct-acting antivirals (DAAs), mass screening, and strong political and public health commitment. The LPS biosynthesis dominance in HCV-HCC corroborates findings of (Dapito et al. 2012), linking endotoxin production to hepatocarcinogenesis (Dapito et al., 2012). In this study, identification of cytochrome P450 homologs as a dominant gene set in HCV-HCC is novel, potentially linked to Egypt's high aflatoxin exposure, a known HCC risk factor which is absent in many global cohorts (Sharaf-Eldin et al., 2016; Omar et al., 2013). The partial restoration of SCFA genes in treated HCV, despite persistent resistance genes, is also a novel finding, suggesting a bifurcated recovery pathway unique to Egyptian patients post-DAA therapy (Hsu et al., 2022). These molecular links underscore the microbiome's role in driving inflammation (NF-κB, bile acids) and oncogenesis (LPS, xenobiotic metabolism), with SCFA restoration as a possible therapeutic target (Tripathi et al., 2018; Schnabl and Brenner, 2014).

The presence of cytochrome P450 homologs, likely of microbial origin, may contribute to aflatoxin detoxification, generating reactive intermediates that cause DNA damage, as observed in fungal P450 systems (Syed and Yadav, 2012). This novel observation warrants validation through targeted metabolomics to detect aflatoxin metabolites in HCC patients, reinforcing the environmental connection to Egypt's HCC burden.

### Microbial biomarkers and clinical translation

HCV-HCC diagnosis relies on Escherichia dominance (Figure 10, LEfSe *p* < 0.05) and cytochrome P450 homolog enrichment (Figure 4), both marking disease-specific signatures with diagnostic potential—corroborated by Ren et al. who identified Escherichia as an HCC biomarker—these drive pathogenesis through LPS biosynthesis (TLR4-mediated gut-liver inflammation) and P450-catalyzed aflatoxin bioactivation (explaining Egypt's unique HCC epidemiology), making them readily targetable via stool qPCR panels for non-invasive surveillance. DAA response monitoring leverages *Lactobacillus* and *Bifidobacterium* recovery (Figures 7, 10) to distinguish treatment responders, alongside partial SCFA pathway restoration that supports adjunctive probiotic/prebiotic interventions to accelerate microbiome normalization. Finally, risk stratification through integrated multi-omics panels combining microbial taxa (*Escherichia*↑, *Faecalibacterium*↓), functional markers (P450, LPS biosynthesis), and host factors (FIB-4 score) enables non-invasive HCC surveillance in high-risk Egyptian cohorts, warranting prospective validation in larger longitudinal studies (Padilha et al., 2025).

### Specific biomarkers and inter-stage links

Taxonomic and functional biomarkers were identified for each stage, with inter-stage links highlighting disease progression as shown in Table 2 below.

**Table 2:** Microbial and molecular signatures across liver disease stages.

Condition	Key bacteria/molecules	Associated genes/pathways	Effects	Figures
Normal	*Faecalibacterium*, Firmicutes, butyrate	Butyrate biosynthesis	Supports gut integrity	13
Liver Disease	Proteobacteria, *Enterobacteriaceae*	NF-κB signaling, bile acid metabolism	Drives inflammation	5, 13
Post-HCV	*Escherichia*, resistance genes	Persistent inflammatory genes	Reflects ongoing dysbiosis	8B, 13
Treated HCV	*Lactobacillus, Bifidobacterium*, acetate	Acetate synthesis	Marks recovery	6, 13
HCV-HCC	*Escherichia*, Proteobacteria, LPS	LPS biosynthesis, cytochrome P450	Promotes oncogenesis	5, 13

The progression of liver diseases reveals a complex interplay of microbial and molecular changes across stages, from healthy states to HCV-related HCC. Escherichia and Proteobacteria emerge as consistent indicators of dysbiosis, linking liver disease to HCV-HCC. Conversely, *Lactobacillus* and SCFA-related genes, particularly those involved in acetate synthesis, connect healthy and treated HCV states, marking a shift toward microbial balance. NF-κB signaling and bile acid metabolism genes, prevalent across diseased stages, drive inflammatory processes, forming a common thread in disease pathology.

The role of Escherichia as an HCC biomarker aligns with (Zhang et al. 2020), affirming its diagnostic potential (Zhang et al., 2020). In contrast, the presence of *Lactobacillus* in treated HCV samples aligns with (Chu et al. 2018), indicating recovery (Chu et al., 2018). Bile acid metabolism genes, prominent in liver disease, corroborate findings of (Zheng et al. 2017), yet their heightened presence in Egyptian patients suggests a unique, HCV-specific dysbiosis, possibly shaped by regional factors (Wang et al., 2022). The persistence of resistance genes in post-HCV samples, as reported by (El-Sokkary et al. 2021), challenges assumptions of full recovery post-viral clearance, indicating a complex recovery process (El-Sokkary et al., 2021).

In healthy controls, the dominance of *Faecalibacterium*-specific butyrate synthesis genes is a novel finding, likely tied to Egypt's distinct dietary habits (Miquel et al., 2013). The identification of cytochrome P450 genes as a novel HCC biomarker may reflect environmental toxin exposure unique to Egypt (Syed and Yadav, 2012). HCV-HCC samples exhibited significant enrichment of microbial cytochrome P450 homologs within xenobiotic metabolism pathways (Figure 4), likely representing bacterial detoxification enzymes that bioactivate dietary aflatoxins—prevalent in Egypt—into genotoxic metabolites, providing a novel microbiome-environment interaction driving regional HCC burden. In treated HCV, the coexistence of restored acetate synthesis genes and persistent resistance genes reveals a distinct recovery process (Garcia-Mantrana et al., 2018; Ponziani et al., 2018).

These microbial and genetic signatures hold significant diagnostic promise. Escherichia and LPS biosynthesis genes emerge as key indicators for detecting HCC, while *Lactobacillus* serves as a beacon for monitoring treatment response (Zhou et al., 2020). Together, these findings paint a vivid picture of the gut-liver axis, where microbial players and their molecular tools shape the trajectory of disease and recovery, offering new strategies for diagnosis and intervention.

### Distinct biomarkers in Egyptian liver patients

*Faecalibacterium* predominantly characterizes the gut microbiome in healthy individuals in this study. This stands in noticeable contrast to many Western studies, where Bacteroides often takes center stage, really underscoring how much a diet could shape these microbial communities (Wu et al., 2011; De Filippo et al., 2010).

Considering liver disease, the presence of genes related to bile acid metabolism isn't entirely surprising, as this aligns with what Ridlon and colleagues discussed back in 2014 (Ridlon et al., 2014). However, what's particularly striking here is how prominent these genes are in the Egyptian context of liver disease. This distinction seems to be closely linked to the significant epidemiological challenge posed by the Hepatitis C Virus (HCV) in Egypt. The post-HCV stage is the discovery of resistance genes that persist even after treatment, which wasn't expected.

In cases of HCV-HCC, the appearance of LPS genes is consistent with what Grat and others reported in 2016 (Grat et al., 2016). But the story gets more interesting with the addition of cytochrome P450 genes to this picture; that's a new piece of the puzzle. Pinpointing cytochrome P450 genes as a biomarker for HCV-HCC primarily for identification, early detection, and potentially prognostic evaluation is a major new insight from this study. The findings could reflect unique environmental factors in Egypt, such as exposure to aflatoxins, which aren't typically a major consideration in HCC studies elsewhere in the world (El-Serag, 2011).

Then, for patients who have been treated for HCV, the emergence of *Bifidobacterium* acetate genes is another novel observation. This could be pointing toward a specific recovery pathway triggered by DAA treatment (Ponziani et al., 2019; Li et al., 2016).

Ultimately, these distinct biomarkers could support accurate diagnosis of these conditions in Egyptian patients. This could pave the way for more personalized screening approaches, especially in regions where HCV is particularly prevalent (Fukui, 2015).

## Conclusions

Shotgun metagenomics revealed progressive dysbiosis across Egyptian liver disease stages, with HCV-HCC characterized by Escherichia/Proteobacteria dominance, cytochrome P450/LPS pathway enrichment, and lowest diversity (*p* < 0.05)—contrasting *Faecalibacterium*-enriched healthy/treated-HCV microbiomes. Novel findings include Egypt-specific cytochrome P450 homologs (aflatoxin bioactivation) and persistent post-DAA resistance genes. Escherichia and P450 genes offer stool-based HCC diagnostic potential; *Faecalibacterium*/SCFA restoration suggests probiotic adjuncts. Limitations include modest sample size (*n* = 46) and lack of dietary metadata. Also, samples were collected without chemical preservatives which could lead to microbial proliferation and a limitation of the study. However, all samples were collected and processed under the same protocol preserving the validity and analysis of the study. Future longitudinal/multi-omics studies will validate these region-specific biomarkers for personalized HCC surveillance and intervention in Egypt's high-risk population.

## Data Availability

The data presented in the study are deposited in the NCBI SRA Repository, accession number: PRJNA1454966.
