# Supplementary material for: Metagenomic profiling of gut microbiome signatures across liver disease stages and HCV-related hepatocellular carcinoma in Egyptian patients
Source: Front Microbiol. 2026 Apr 28;17:1758563. doi: 10.3389/fmicb.2026.1758563 (PMC13163684; doi:10.3389/fmicb.2026.1758563)
Supplement: Supplementary file 1 [file Supplementary_file_1.docx]

**Supplementary 1:** **Venn Diagram of unique gene sets for stage-specific groups**. Venn diagram showing overlap and unique gene sets among normal, liver disease, Post-HCV, Treated-HCV, and HCV-HCC groups from metagenomic sequencing. This comparative analysis highlights both shared molecular signatures and condition-specific gene expression patterns associated with liver disease progression and hepatitis C virus (HCV)-related hepatocellular carcinoma (HCC).


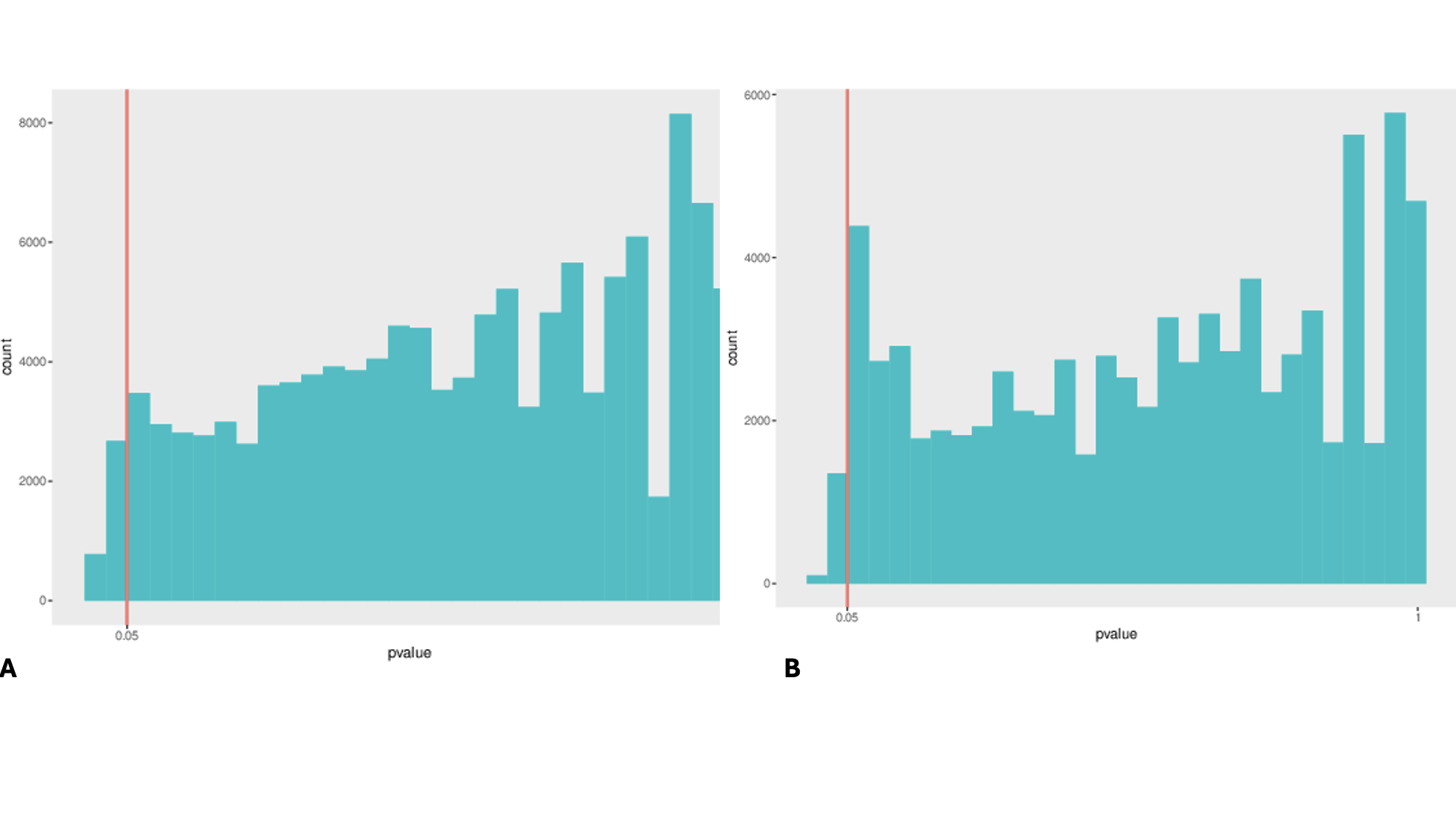


**Supplementary 2:** **Evaluation of differential abundant genes pairwise amongst groups via p-value distribution.** Histograms depict the distribution of p-values obtained from statistical tests assessing differential significance across two datasets. The red vertical line indicates the conventional significance threshold show a relatively uniform pattern with a modest accumulation of low p-values near the significance threshold. (A). Distribution for differentially abundant genes between post-HCV and HCV-HCC samples. (B). Distribution of p-value for differentially abundant genes between post-HCV and treated HCV samples demonstrates a near-uniform pattern but with a slightly higher proportion of tests below the p = 0.05 threshold.


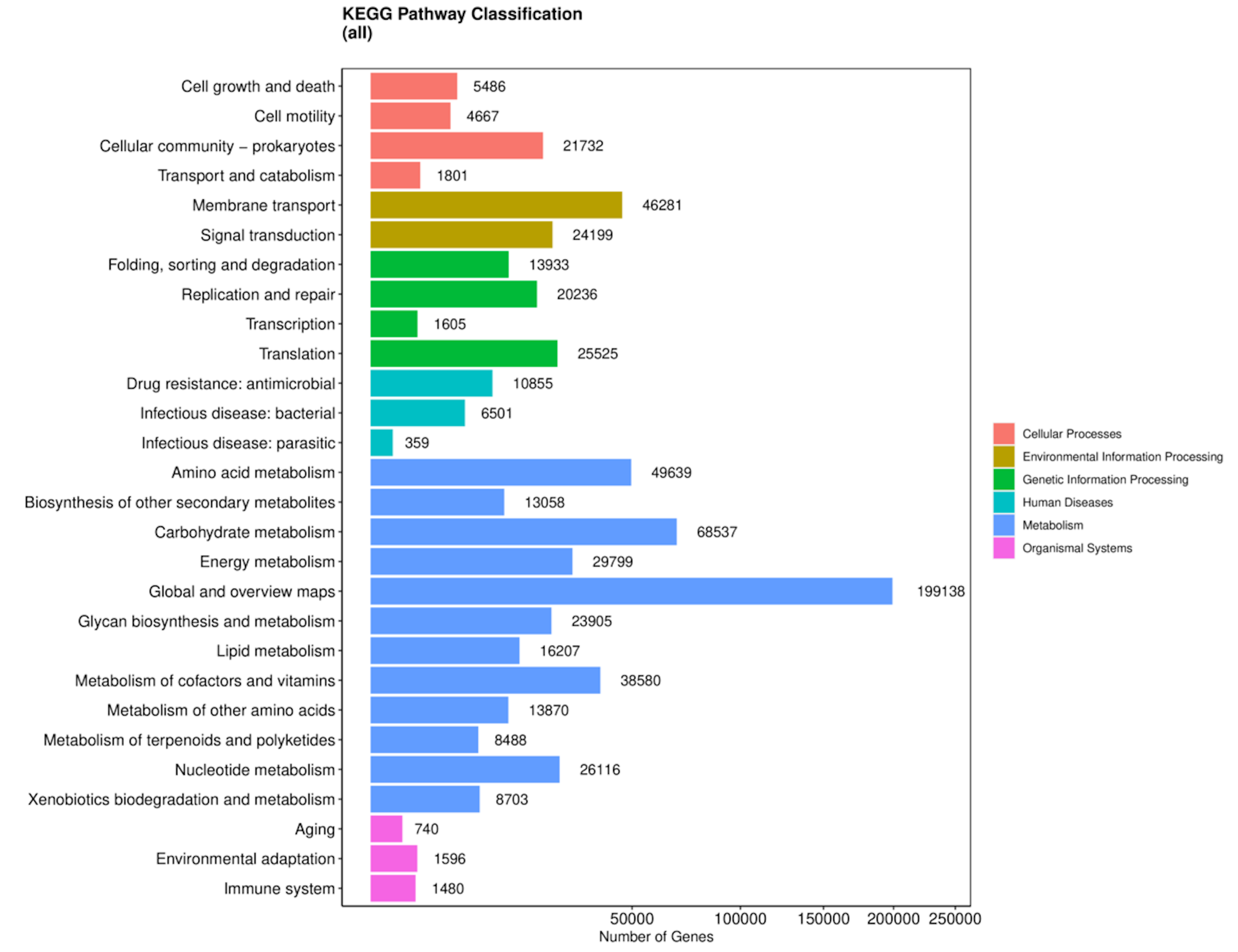


**Supplementary 3**: **KEGG Pathway Classification of Annotated Genes for All Groups.** Barplot summarizing KEGG orthology (KO) terms across all groups, categorized by function (e.g., metabolism, genetic information processing). Metabolic pathways, particularly **Global and overview maps** (199,138 genes) and **Carbohydrate metabolism** (68,537 genes), accounted for the largest proportion of annotated genes, reflecting a strong enrichment in metabolic activities. In contrast, pathways such as **Aging** (740 genes) and **Infectious disease: parasitic** (359 genes) were represented by relatively few genes. Overall, the classification highlights that the majority of annotated genes are involved in metabolic and genetic information processing functions.


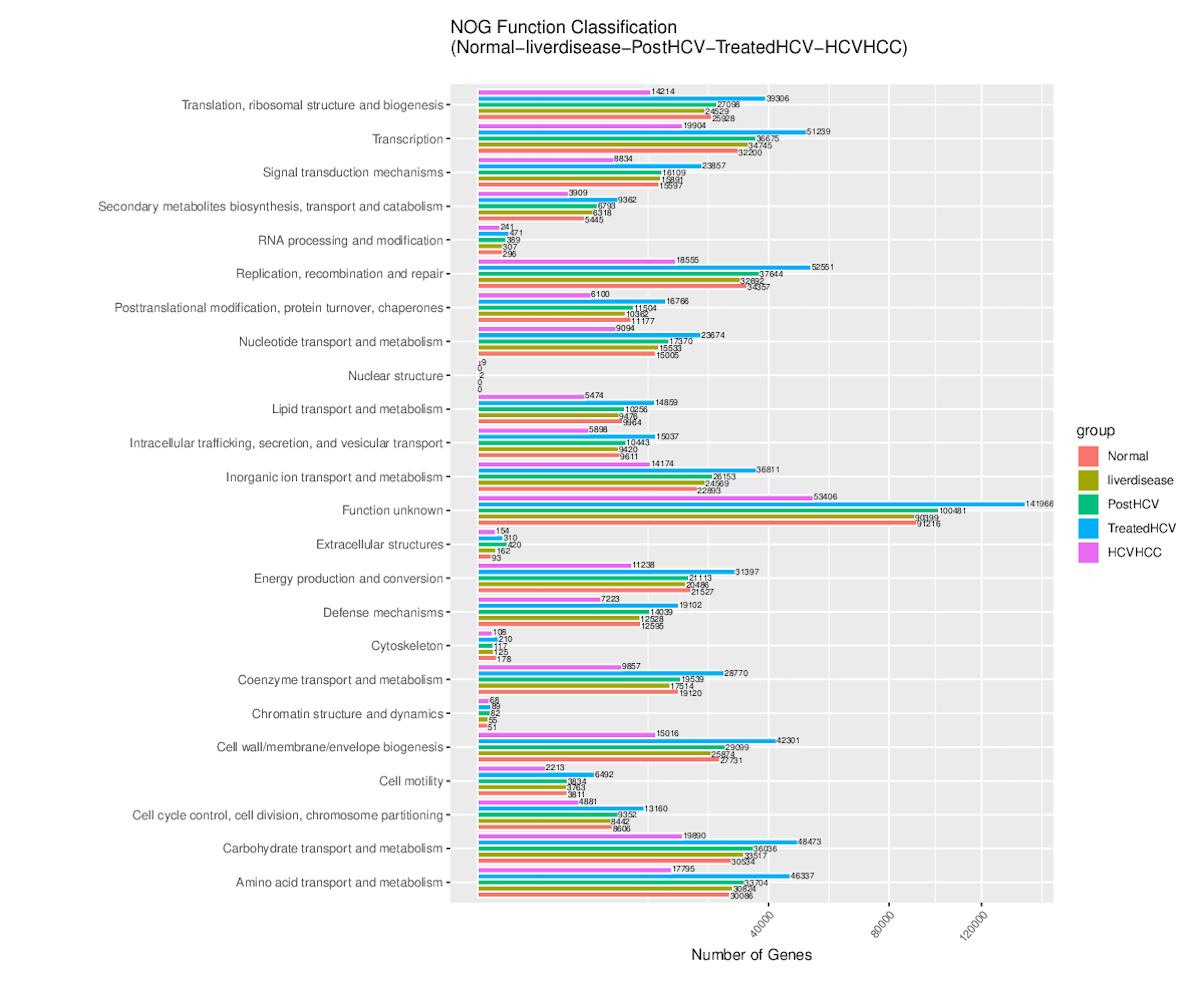


**Supplementary 4**: **eggNog functional classification of gut microbiome genes across different liver disease stages in Egyptian patients.** This bar plot visually represents the distribution of predicted microbial gene functions across the five study groups. Distinct shifts in functional gene abundance are observed between disease stages, there are higher representation of genes involved in energy production, amino acid metabolism, and membrane biogenesis in HCVHCC and treated HCV groups which suggest microbial adaptation to host disease progression and antiviral treatment.

**Supplementary Table 1: Shotgun Metagenomic Assembly Statistics Across Disease Groups.** Comprehensive assembly metrics from MEGAHIT v1.0.2 de novo assembly of clean reads (post-SOAPnuke QC) across 46 Egyptian patient samples. N50/N90 values confirm adequate contig length for gene prediction (MetaGeneMark); mapping rates (70-91%) validate high-quality clean read recovery. HCC samples maintain comparable assembly quality to controls despite disease state.

| **Sample** | **Contig Number** | **Assembly Length (bp)** | **N50 (bp)** | **N90 (bp)** | **Max (bp)** | **Min (bp)** | **Average Size (bp)** | **Mapping Rate(%)** |
| --- | --- | --- | --- | --- | --- | --- | --- | --- |
| AIH10 | 54051 | 145854747 | 8324 | 964 | 513183 | 300 | 2698 | 77.19 |
| AIH21 | 36905 | 93995938 | 14308 | 800 | 597863 | 300 | 2546 | 84 |
| AIH31 | 7290 | 31556835 | 53246 | 1319 | 773645 | 300 | 4328 | 87.68 |
| CH8 | 11298 | 31917405 | 11099 | 947 | 557578 | 300 | 2825 | 86.84 |
| CH25 | 58732 | 133216580 | 6151 | 794 | 439639 | 300 | 2268 | 78.04 |
| CH33 | 35665 | 79898768 | 6059 | 780 | 357947 | 300 | 2240 | 78.52 |
| HBV5 | 55325 | 117175188 | 5289 | 749 | 497693 | 300 | 2117 | 79.41 |
| HBV9 | 65879 | 137532491 | 5653 | 726 | 754229 | 300 | 2087 | 77.12 |
| N1 | 54994 | 159732943 | 11506 | 975 | 414331 | 300 | 2904 | 78.23 |
| N2 | 61834 | 164573282 | 8600 | 900 | 446102 | 300 | 2661 | 78.64 |
| N3 | 68626 | 157598439 | 6343 | 797 | 897080 | 300 | 2296 | 79.57 |
| N4 | 55763 | 135948832 | 6976 | 849 | 962517 | 300 | 2437 | 79.68 |
| N5 | 48106 | 168212756 | 19045 | 1133 | 736317 | 300 | 3496 | 80.83 |
| N6 | 43686 | 163584943 | 22073 | 1221 | 736829 | 300 | 3744 | 81.31 |
| N7 | 27327 | 85718658 | 17064 | 1019 | 946801 | 300 | 3136 | 82.23 |
| N8 | 57401 | 152326035 | 7525 | 938 | 392388 | 300 | 2653 | 79.07 |
| N9 | 17368 | 41767045 | 8600 | 826 | 946801 | 300 | 2404 | 80.47 |
| N10 | 29537 | 90953971 | 11936 | 1060 | 279554 | 300 | 3079 | 82.84 |
| pHCV1 | 21278 | 64926978 | 11405 | 1044 | 917449 | 300 | 3051 | 84.29 |
| pHCV2 | 49246 | 69258585 | 2721 | 484 | 791893 | 300 | 1406 | 83.18 |
| pHCV6 | 11170 | 47201172 | 17931 | 1563 | 552700 | 300 | 4225 | 85.22 |
| pHCV12 | 11966 | 37286205 | 10461 | 1055 | 523109 | 300 | 3116 | 80.21 |
| pHCV15 | 30692 | 102750204 | 16911 | 1131 | 604763 | 300 | 3347 | 82.55 |
| pHCV18 | 20867 | 67309996 | 28885 | 1014 | 532750 | 300 | 3225 | 84.57 |
| pHCV19 | 40729 | 122425255 | 14162 | 1028 | 604763 | 300 | 3005 | 81.95 |
| pHCV22 | 70071 | 125481217 | 4269 | 613 | 291615 | 300 | 1790 | 79.01 |
| pHCV27 | 42960 | 80521930 | 7103 | 580 | 526443 | 300 | 1874 | 82.01 |
| pHCV28 | 47815 | 69440501 | 2838 | 506 | 336269 | 300 | 1452 | 81.66 |
| pHCV29 | 62965 | 154488549 | 6632 | 887 | 517809 | 300 | 2453 | 75.52 |
| pHCV30 | 33256 | 106083501 | 11233 | 1119 | 707339 | 300 | 3189 | 79.61 |
| tHCV11 | 29280 | 93297789 | 8963 | 1147 | 831246 | 300 | 3186 | 82.97 |
| tHCV13 | 48509 | 165151555 | 15475 | 1157 | 1045152 | 300 | 3404 | 75.51 |
| tHCV14 | 36994 | 78893617 | 8267 | 666 | 355191 | 300 | 2132 | 82.03 |
| tHCV23 | 24510 | 83098937 | 19356 | 1140 | 631615 | 300 | 3390 | 82.41 |
| tHCV32 | 4246 | 17169901 | 25525 | 1279 | 249741 | 300 | 4043 | 91 |
| tHCV34 | 113952 | 193307336 | 3893 | 574 | 302506 | 300 | 1696 | 71.48 |
| tHCV38 | 43875 | 96126175 | 5542 | 774 | 237755 | 300 | 2190 | 76.01 |
| tHCV39 | 109774 | 203843182 | 4193 | 652 | 634384 | 300 | 1856 | 76.86 |
| tHCV40 | 98479 | 196263772 | 3954 | 749 | 347419 | 300 | 1992 | 76.93 |
| tHCV42 | 89938 | 225737120 | 6248 | 926 | 844998 | 300 | 2509 | 69.7 |
| HCC3 | 24502 | 48337514 | 4254 | 744 | 437200 | 300 | 1972 | 83.2 |
| HCC4 | 20796 | 54428429 | 6412 | 970 | 655149 | 300 | 2617 | 85.31 |
| HCC20 | 29014 | 83228426 | 9854 | 1009 | 532858 | 300 | 2868 | 81.58 |
| HCC24 | 33687 | 76062642 | 9781 | 733 | 544984 | 300 | 2257 | 82.25 |
| HCC26 | 45270 | 101589553 | 6192 | 781 | 293074 | 300 | 2244 | 81.82 |
| HCC41 | 23417 | 49771877 | 4748 | 751 | 428209 | 300 | 2125 | 86.67 |
